# Local adaptation of *Dromiciops* marsupials (Microbiotheriidae) from southern South America: Implications for species management facing climate change

**DOI:** 10.1002/ece3.70355

**Published:** 2024-10-03

**Authors:** Julian F. Quintero‐Galvis, Pablo Saenz‐Agudelo, Guillermo D'Elía, Roberto F. Nespolo

**Affiliations:** ^1^ Instituto de Ciencias Ambientales y Evolutivas Universidad Austral de Chile Valdivia Chile; ^2^ Millennium Nucleus of Patagonian Limit of Life (LiLi) Valdivia Chile; ^3^ Millenium Nucleus for Ecology and Conservation of Temperate Mesophotic Reefs (NUTME) Las Cruces Chile; ^4^ Colección de Mamíferos Universidad Austral de Chile Valdivia Chile; ^5^ Center of Applied Ecology and Sustainability (CAPES), Facultad de Ciencias Biológicas Universidad Católica de Chile Santiago Chile; ^6^ Millennium Institute for Integrative Biology (iBio) Santiago Chile

**Keywords:** Chile, local adaptation, marsupials, monito del monte, population genomic, Valdivian Forest

## Abstract

The two species of the microbiotheriid marsupial genus *Dromiciops* (*Dromiciops bozinovici*: “Panchos's monito del monte” and *Dromiciops gliroides*: “monito del monte”) exhibit a marked latitudinal genetic differentiation. Nevertheless, it is unclear whether this differentiation results from neutral processes or can be explained, to some extent, by local adaptation to different environmental conditions. Here, we used an SNP panel gathered by Rad‐seq and searched for footprints of local adaptation (putative loci under selection) by exploring genetic associations with environmental variables in the two species of *Dromiciops* in Chilean and Argentinean populations. We applied three methods for detecting outlier SNPs and two genotype–environment associations approaches to quantify associations between allelic frequencies and environmental variables. Both species display strong genetic structure. *D*. *bozinovici* exhibited three distinct genetic groups, marking the first report of such structuring in this species using SNPs. In contrast, *D*. *gliroides* displayed four genetic clusters, consistent with previous studies. Both species exhibited an association of their genetic structure with environmental variables. *D*. *bozinovici* exhibited significant associations of allelic frequencies with elevation, precipitation during the warmest periods, and seasonality in the thermal regime. For *D*. *gliroides*, genetic variation appeared to be associated with more variables than *D*. *bozinovici*, including precipitation and temperature‐related variables, isothermality, and elevation. All the outlier SNPs were mapped to the *D*. *gliroides* reference genome to explore if they fell within functionally known genes. These results represent a necessary first step toward identifying the genome regions that harbor genes associated with climate adaptations in *Dromiciops*. Notably, we identified genes involved in various functions, including carbohydrate synthesis (ALG8), muscle and neuronal regulation (MEF2D), and stress responses (PTGES3). Ultimately, this study contributes valuable insights that can inform targeted conservation strategies aimed at preserving the genetic diversity of *Dromiciops* in the face of environmental challenges.

## Graphical abstract

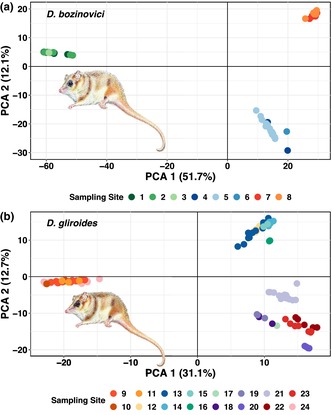

## 1 INTRODUCTION

Uncovering how environmental heterogeneity contributes to spatially structured genomic variation can shed light on how local adaptation allows species to establish in diverse habitats with regionally distinct selection regimes (Prates et al., 2018). To directly test the occurrence of local adaptation requires common‐garden or reciprocal transplant experiments and fitness estimates that allow the dissection of environmental and genetic effects (Savolainen et al., 2013; Tigano et al., 2017). These experiments are difficult to implement due to distinct external factors (e.g., logistics, ethics, and the life‐history characteristics of many species; Tigano et al., 2017). When these experiments are not possible, alternative approaches using other proxies, such as comparing allele frequencies of populations distributed across environmental gradients or patchy environments are used (Rellstab et al., 2015; Savolainen et al., 2013; Tigano et al., 2017). Population genetic theory predicts that genes related to traits under selection due to different environmental conditions are expected to display specific changes in allele frequencies compared to alleles of genes not under selection (Dauphin et al., 2023; Dudaniec et al., 2018). For example, clinal variation in allele frequencies has been associated with environmental factors, such as altitude in amphibians (Bonin et al., 2006), latitude in butterflies and lizards (De Jong et al., 2013; Prates et al., 2018), precipitation in tropical birds (Termignoni‐García et al., 2017), and water availability in Patagonian rodents (Giorello et al., 2018). In turn, characterizing patterns of selection and local adaptation is relevant to design management and conservation strategies since relocating animals successfully depends on knowledge of the population's genetic architecture and how it allows organisms to survive in a given environment (Hoffmann & Sgrò, 2011).

Current sequencing technologies have revolutionized genomic studies of non‐model species by allowing access to panels of thousands of SNPs. Analytical advancements allow for tackling a broad range of evolutionary and ecological questions (Waldvogel et al., 2020). While technologies have indeed reduced costs, certain limitations persist in the analysis of whole‐genomes or transcriptomes, as gathering these types of data is still relatively expensive when comparing several populations and individuals (da Fonseca et al., 2016). Alternatively, restriction site‐associated DNA sequencing (Rad‐seq), which consistently scans a small proportion of the genome, represents a reliable, replicable, and low‐cost alternative (Baird et al., 2008). The high‐throughput nature of the data gathered through Rad‐seq allows robust analyses for a variety of questions, including quantitative trait loci analysis, evaluation of genetic adaptations to environmental conditions, accurate phylogeographic inferences (Rochette & Catchen, 2017), and the characterization of allele frequency variation in relation to environmental gradients (Tigano et al., 2017).

The genus *Dromiciops* provides an interesting case study to explore local adaptation using genomic tools. Over the past decade, there has been ongoing debate regarding the number of species within the genus *Dromiciops*. In 2016, D'Elía et al. (2016) proposed that the genus was polytypic, consisting of three species. This was a shift from the prior understanding that recognized only a single species (*Dromiciops gliroides*). Following this proposal, several studies suggested the genus was monotypic, supporting the recognition of only one species (Martin, 2019; Suárez‐Villota et al., 2018; Valladares‐Gómez et al., 2017; see a critique on the merits of these studies in D'Elía et al., 2020). Currently, in the light of all available evidence, including DNA gene sequence and genomic data, the consensus is that *Dromiciops* is not monotypic. Some taxonomies recognize two species: *Dromiciops bozinovici* and *D*. *gliroides*, with the latter composed of two subspecies: *D*. *g*. *gliroides* and *Dromiciops g*. *mondaca* (Quintero‐Galvis et al., 2021, 2022); while other taxonomic schemes consider three species, with *D*. *mondaca* considered at the species level (Astúa et al., 2022; D'Elía et al., 2020). Species of *Dromiciops* live in the Valdivian rainforest. The distributional range of *D*. *bozinovici* is restricted between the Maule and Araucanía regions of Chile and adjacent areas of the Argentinean province of Neuquén (between 36° S and 39° S; D'Elía et al., 2016). The distributional range of *D*. *gliroides* is to the south of that of *D*. *bozinovici*, between 39′3° S and 45° S in Chile and Argentina (D'Elía et al., 2016; Oda et al., 2019). Climate change predictions for the Valdivian rainforest region indicate increased temperatures and altered precipitation patterns, which are expected to impact local ecosystems significantly (Muñoz‐Sáez et al., 2021). Predicted climate impacts in this area include shifts in species distributions and changes in habitat suitability for the species of *Dromiciops* (Vazquez et al., 2022). These climate‐associated changes pose management challenges, such as planning habitat corridors to facilitate species movement and adapting conservation plans to prevent biodiversity loss and ensure ecosystem resilience. Understanding the climate‐associated adaptive divergence in species of *Dromiciops* is crucial for developing effective conservation strategies. Effective strategies could include habitat protection, assisted gene flow to boost genetic diversity, and potentially relocating individuals or populations to areas predicted to remain viable under future climate conditions (as suggested by Chen et al., 2022; Gaitán‐Espitia & Hobday, 2021). The environmental conditions across these territories vary both within and between species. For example, populations of *D*. *bozinovici* found in the “Maulino” forest or exotic plantations in the Maule and Bio‐Bio Regions (Cortez Parra & Torres‐Fuentes, 2021; Uribe et al., 2017) are exposed to a significantly drier environment than southwards of 39° S distribution in the Araucania region. This fact is relevant since niche modeling studies have suggested that precipitation and some temperature‐related variables are the best explanators for the presence of *Dromiciops* across its range (Martin, 2010; Vazquez et al., 2022). However, no study has correlated genetic and environmental variation. Interestingly, Quintero‐Galvis et al. (2022) using SNP data, described a deep genetic structure for *D*. *gliroides* but low genetic variability for *D*. *bozinovici*. Here, we evaluate if there are genomic signatures of local adaptation (putative loci under selection) in the two species of *Dromiciops* and test if putative loci under selection are associated with environmental variables. We hypothesize that *D*. *bozinovici* and *D*. *gliroides* exhibit a small degree of ecological adaptation across their respective distribution ranges, with specific allelic frequencies correlated with environmental factors. To test this, we perform a study of genomic‐environmental association by incorporating additional individuals/SNPs into a new SNP data set originally reported by Quintero‐Galvis et al. (2022) to evaluate whether the pattern of genomic variation is associated with environmental variation. In addition, we performed a redundancy analysis (RDA) to correlate genomic data variation with environmental factors. Our final aim was to use genomic information to understand how these species might be vulnerable to climate change. We modeled the rate of past evolutionary change to predict future genomic vulnerability based on the allelic frequency of candidate loci. These findings help predict how climate change could impact genetic diversity and adaptation capacities, and can thus, inform strategies for managing and relocating individuals or populations in response to climate change.

## 2 METHODS

**FIGURE 1:**
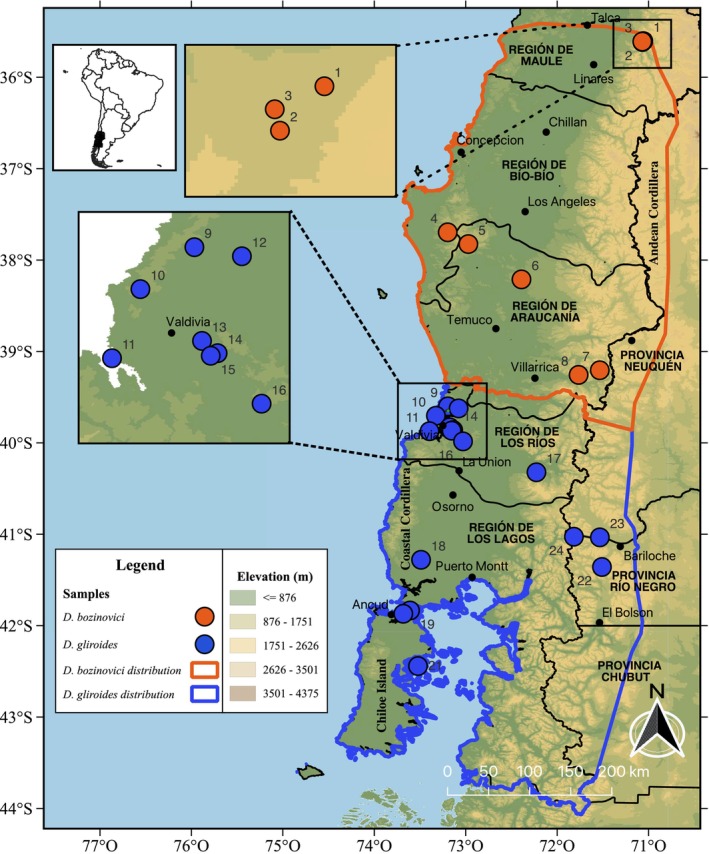
Map showing the distribution of *Dromiciops bozinovici* and *D*. *gliroides*. Numbers correspond to sampling localities as indicated in Table 1.

Fieldwork was carried out in the austral 2022–2023 summer. We sampled eight localities for *D*. *bozinovici* and 16 localities for *D*. *gliroides* in total (Table 1; Figure 1). Animals were live trapped in each location using tomahawk traps during nighttime; a small skin clip was taken from one ear of each individual, which was immediately released at the capture place. The entire procedure was performed according to the bioethical regulations of Universidad Austral de Chile (UACh; license 313 issued to Roberto F. Nespolo and license 455 issued to Julian F. Quintero‐Galvis) and collection permits granted by the national authority Servicio Agricola Ganadero (permits 4371 and 3393). Ear samples were preserved in 96% ethanol, and DNA was extracted using the GenJet Genomic DNA kit (Thermo Fisher Scientific). DNA quality was checked by visualization on a 0.7% agarose gel, and DNA concentration was measured using the Qubit™ dsDNA HS Assay Kit and the Life Technologies Qubit fluorometry system (Thermo Fisher Scientific). All samples were normalized to a final concentration of 18 ng/μl. Library preparation and RAD sequencing from genomic DNA were performed by Floragenex using the restriction enzyme *SbfI* and sample‐specific barcodes following the protocol described by Baird et al. (2008). All raw fastq files are available at NCBI under BioProject accession number PRJNA108845.

**TABLE 1:** Summary statistics for the analyzed sampling localities of *Dromiciops bozinovici* and *D*. *gliroides*.

Species	Site	Maps	Country	SC	*N*	Latitude	Longitude	Elevation	Cluster	Ho	He	Fis	*π*	Data source
*Dromiciops bozinovici*	Altos de Lircay Alto	1	Chile	ALCH	12	−35.604	−71.058	1344	DB‐A	0.00941	0.01137	0.00522	0.0003	This study
	Altos de Lircay Bajo	2	Chile	ABCH	6	−35.614	−71.068	1301	DB‐A	0.00778	0.01034	0.00545	0.0002	This study
	Tacitas	3	Chile	TTCH	1	−35.609	−71.069	1277	DB‐A	0.00916	–	–	0.0002	This study
	Caramavida	4	Chile	CACH	2	−37.696	−73.197	863	DB‐B	0.09233	0.13643	0.03745	0.0029	Quintero‐Galvis et al. (2022)
	Nahuelbuta	5	Chile	NACH	22	−37.825	−72.971	1184	DB‐B	0.03883	0.14267	0.30788	0.0034	Quintero‐Galvis et al. (2022)
	Victoria	6	Chile	AACH	1	−38.211	−72.390	355	DB‐B	0.06279	–	–	0.0015	This study
	Pucon	7	Chile	PCCH	19	−39.205	−71.534	1226	DB‐C	0.01217	0.15507	0.10082	0.0038	This study
	KodKod	8	Chile	KTCH	7	−39.256	−71.766	530	DB‐C	0.01139	0.14891	0.07673	0.0036	This study
*Dromiciops gliroides*	San Martin	9	Chile	SMCH	27	−39.600	−73.194	36	DG‐A	0.06999	0.13992	0.21521	0.0014	Quintero‐Galvis et al. (2022)
	Parque Oncol	10	Chile	ONCH	13	−39.703	−73.326	568	DG‐A	0.06613	0.13315	0.1788	0.0013	Quintero‐Galvis et al. (2022)
	Niebla	11	Chile	NNCH	2	−39.872	−73.396	107	DG‐A	0.07168	0.1301	0.05223	0.0011	This study
	Pelchuquin	12	Chile	PACH	3	−39.622	−73.078	202	DG‐A	0.08024	0.13546	0.07616	0.0012	This study
	Forestal Calle Calle	13	Chile	CCCH	8	−39.830	−73.175	188	DG‐B	0.05584	0.12432	0.15606	0.0012	Quintero‐Galvis et al. (2022)
	Parque Llancahue	14	Chile	PLCH	20	−39.860	−73.137	348	DG‐B	0.06924	0.12563	0.16671	0.0012	Quintero‐Galvis et al. (2022)
	Donoso	15	Chile	DDCH	4	−39.866	−73.154	164	DG‐B	0.10757	0.12558	0.03199	0.0012	Quintero‐Galvis et al. (2022)
	Caman, Paillaco	16	Chile	RCCH	2	−39.983	−73.030	423	DG‐B	0.09424	0.1204	0.0245	0.0011	This study
	Rininahue, Lago Ranco	17	Chile	FFCH	2	−40.321	−72.227	110	DG‐B	0.08002	0.05948	0.01905	0.0007	This study
	Fresia, Los Muermos	18	Chile	MMCH	1	−41.279	−73.489	136	DG‐C	0.03581	–	–	0.0003	This study
	Caulin Chiloé	19	Chile	CDCH	3	−41.833	−73.609	30	DG‐C	0.01156	0.04446	0.02688	0.0003	Quintero‐Galvis et al. (2022)
	Senda Darwin	20	Chile	SDCH	4	−41.869	−73.683	41	DG‐C	0.00859	0.0504	0.04165	0.0003	Quintero‐Galvis et al. (2022)
	Quinchao, Chiloé	21	Chile	CLCH	4	−42.441	−73.522	16	DG‐C	0.02532	0.03772	0.01472	0.0004	This study
	Laguna Verde	22	Argentina	PBAG	16	−41.357	−71.510	1023	DG‐D	0.03888	0.12405	0.22073	0.0012	Quintero‐Galvis et al. (2022)
	Bariloche	23	Argentina	LAAG	5	−41.033	−71.533	769	DG‐D	0.04357	0.0823	0.07156	0.0008	Quintero‐Galvis et al. (2022)
	Puerto Blest	24	Argentina	LVAG	7	−41.026	−71.817	973	DG‐D	0.03736	0.07483	0.07974	0.0007	Quintero‐Galvis et al. (2022)

*Note*: Maps correspond to sites in Figure 1. Elevation (meters above sea level (m.a.s.l.)). Clusters correspond to genetic groups by species.

Abbreviations: F_IS_, Inbreeding coefficient; He, Expected heterozygosity; Ho, Observed heterozygosity; *N*, Number of specimens per site and genetic diversity (*π*); SC, Site code.

### 2.1 RAD sequencing

Details about library preparation, sequencing, and bioinformatic parameters used to identify variable loci were described in Quintero‐Galvis et al. (2022). Briefly, libraries were prepared using a Rad‐seq protocol (Baird et al., 2008) and then sequenced using 2 × 100 bp sequencing on seven lanes of an Illumina HiSeq 2500. The dataset was compiled and analyzed using the STACKS v2 bioinformatics pipeline (Catchen et al., 2011; Rochette et al., 2019) for SNP detection and assembly with the program “ref_map.pl.” Demultiplexed reads were aligned to the reference genome of *D*. *gliroides* (Feng et al., 2022) using Bowtie2 (Langmead & Salzberg, 2012) with “‐sensitive” settings.

Aligned read files were then used to generate a catalog of RAD loci using ref_map.pl. in STACKS v2. We then used the population's module in Stacks to calculate population genetic statistics and filter loci. We kept only loci present in at least 95% of the samples, with a minor allele frequency (MAF) of at least 2% and a maximum observed heterozygosity of 0.6. Establishing a heterozygosity limit helps to eliminate loci with excess heterozygosity, which may indicate alignment errors or duplicated loci, thereby ensuring the reliability and representativeness of the genetic diversity data. The MAF threshold of 2% helps to exclude extremely rare variants, reducing the inclusion of sequencing artifacts while retaining biologically relevant genetic variations present at a low frequency. Only one SNP per locus was kept using the flag “write‐random‐snp.” Analyses were based on the variant call format (VCF) file with SNP per locus using STACKS v2 (Catchen et al., 2011, 2013; Rochette et al., 2019). PGDSpider v2.1 software (Lischer & Excoffier, 2012) was used to convert the VCF into specific input files.

### 2.2 Population genetic diversity and structure

Basic population genetic statistics (i.e., SNP nucleotide diversity [*π*] and inbreeding coefficients [F_IS_]) were generated with STACKS (Catchen et al., 2011) for each locality of each species. We used the summary function in the *adegenet* package (Jombart, 2008) in R v3.6 (R Core Team, 2023) to generate observed and expected heterozygosity estimates. For each species, we ran a landscape and ecological association analysis (LEA; Frichot & François, 2015) to estimate the most likely number of genetic clusters by running the snmf algorithm for values of K = 1 to K = 10. To visualize the distribution of the genetic variation among individual specimens, we performed a principal component analysis (PCA) with the *dudi*.*pca* function from the ade4 package (Dray & Dufour, 2007). A hierarchical analysis of molecular variance (AMOVA) was conducted to measure population differentiation among sites, and the genetic groups identified with the LEA and PCA (i.e., grouping by genetic clusters) was performed with the function *poppr*.*amova* in the *poppr* package (Kamvar et al., 2014) in R (R Core Team, 2023).

### 2.3 Data raster

We utilized the WorldClim database (Fick & Hijmans, 2017), which includes 19 bioclimatic variables. For each locality of each species under study, we extracted the climatic variable values from the respective layers within the WorldClim database. Variables were retrieved in the projected latitude/longitude coordinate reference system (WGS84 datum). Altitude information was retrieved from the SRTM 90m Digital Elevation Database (Jarvis et al., 2008), which provides 5 × 5 degree tiles covering Earth's land surface in the latitude/longitude coordinate reference system (WGS84 horizontal datum, EGM96 vertical datum), with a 90 m^2^ resolution at the Equator. Quantum GIS (v.3.4) (QGIS Development Team, 2020) was used to carry out operations on raster files (Figure 1). To avoid high collinearity, per highly correlated variables (|*r*| > .8), we only use one of them, removing the others. This criterion ensured that the retained variables were statistically appropriate and biologically meaningful. For *D*. *bozinovici*, four climatic variables and elevation were kept for subsequent analysis: bio4 (temperature seasonality), bio5 (maximum temperature of the warmest month), bio11 (Mean Temperature of the coldest quarter), and bio18 (Precipitation of the warmest quarter). For *D*. *gliroides*, seven climatic variables remained for subsequent analysis: bio1 (Annual mean temperature), bio3 (Isothermality), bio4 (temperature seasonality), bio10 (Mean Temperature of Warmest Quarter), bio15 (Precipitation seasonality), bio18 (Precipitation of the warmest quarter), bio19 (Precipitation of the coldest quarter), and elevation.

### 2.4 Isolation by distance and isolation by environment

To investigate the role of geographic and environmental factors in shaping spatial genetic differentiation, we assessed the existence of (a) isolation by distance (IBD), (b) isolation by environment (IBE) patterns, and (c) the correlation between environmental and geographic distance among populations. Pairwise population *F*_ST_/(1 − *F*_ST_) was calculated using the R package *hierfstat* (Goudet, 2005). The Mantel test was used to assess associations between linearized *F*_ST_/(1 − *F*_ST_) and geographic and environmental distances; the significance of the associations was tested using 999 permutations in the R package *vegan* (Oksanen et al., 2018). Environmental distances between site pairs were calculated by scaling and centering the selected climatic variables to account for differences in magnitude and then calculating pairwise Euclidean distances. Plots and statistical analyses were conducted using R (R Core Team, 2023).

### 2.5 Signatures of selection and outlier detection

We applied three methods to detect outlier SNPs as implemented in BayeScan v2.1 (Foll & Gaggiotti, 2008), *pcadapt* (Luu et al., 2017), and *fdist2* (Excoffier et al., 2009). These methods were chosen because they have different theoretical assumptions, each prone to different errors. In a conservative approach, only loci identified as outliers by all three methods were considered in further analyses. Analyses were run separately for each species.

BayeScan uses a Bayesian approach via Markov Chain Monte Carlo (MCMC) to consider the uncertainty in allele frequencies, especially when dealing with small sample sizes. This is crucial for accurately estimating posteriors in population genetics analyses. The program calculates prior odds for neutral models by comparing the posterior probability of two models, one with and one without selection on a locus, using the proportion of loci with a strong increase in *F*_ST_ relative to other loci among the MCMC outputs of its simulations (Beaumont & Balding, 2004). The parameters used were 5000 burn‐in iterations, followed by 10,000 iterations with a thinning interval of 10. We considered the Gelman‐Rubin convergence diagnostic from the coda package (Plummer et al., 2006). For this diagnostic, convergence is typically considered acceptable if R̂ (Rhat) is less than 1.1. We used this threshold in our analysis to ensure that our Markov chains had adequately converged. The ratio between the neutral and selection models ranged from 100 to 1000. The posterior odds, or ratio of posterior probabilities, were used to define thresholds to determine sets of outlier markers (Foll & Gaggiotti, 2008). After FDR correction for false positives, a locus was accepted as an outlier if the odds ratio was 0.05 or higher. The R package *pcadapt* uses a PCA approach to estimate the relative contribution of each locus and ultimately identify loci with disproportionate contributions to population structure after controlling by population structure without a priori population definitions. *pcadapt* assumes that the SNPs excessively associated with population structure are candidates for local adaptation—loci in which a qval < alpha (alpha = FDR of 0.05) were accepted as outliers. Values of K were determined from the mean squared error rate for each species following criteria from Evanno et al. (2005). Finally, we used the hierarchical island model as implemented in the *fdist2* function of Arlequin v3.5 (Excoffier & Lischer, 2010). Arlequin uses coalescent simulations to create a null distribution of F statistics to generate the probability distribution for each locus based on the levels of observed heterozygosity and divergence measured as *F*_ST_ (Excoffier et al., 2009). The parameters used to run the software were 100,000 simulations with 1000 demes and 100 repetitions by groups. Loci that fell outside the 99% quantile were considered candidates for directional selection.

### 2.6 Genotype–environment association

Two complementary environmental association (EA) methods were used to identify outliers in the analysis separately for each species. Initially, a RDA was run to estimate how much of the variance in SNP genotypes was correlated to climatic variables and population structure (based on genetic groups identified with the LEA and PCA; see Table 1). The RDA was run using the R package *vegan* (Oksanen et al., 2018). This approach is a multivariate analog of linear regression, which examines how much variation in one set of variables explains the variation in another set (Borcard et al., 1992). This tool applies well to landscape genomics because it can determine how a matrix of landscape variables explains variation in adaptive allele frequencies while overcoming linearity assumptions (Kierepka & Latch, 2015). RDA is a constrained linear ordination method that combines multiple linear regression and PCA. Population structure effects were approximated by the first two principal components of a new PCA performed in the R package *vegan* (Oksanen et al., 2018). We build a vector that accounts for population structure (genetic clusters for each species). Two PCA vectors (PCA1 and PCA2) were analyzed when correlated predictors with |*r*| > .8 were removed. Since spatial autocorrelation is usually reflected by neutral genetic structure, we did not include spatial covariates to avoid over‐conditioning the model (Rellstab et al., 2015). We use a partial RDA to evaluate the effects of environment and genotype variation while controlling for the effect of population structure. The independent effect of the environment was the variance values for the constrained matrix of population structure in the RDA, while the independent effect of population structure was the equivalent for the constrained matrix of climatic variables. Given the significant population structure observed in both species, the RDA includes population structure (Genetic structure) as a covariate. The collinear proportion was calculated by subtracting the environment's independent effects and population structure from the total variance explained in the full RDA model. For identifying candidate SNPs, we used the first two RDA axes, which explained most of the variance (over 70%). Candidate SNPs were identified based on extreme loading values in the RDA ordination space (Forester et al., 2018).

We also ran a latent factor mixed model (LFMM; Frichot et al., 2013) to select outliers with |z|‐scores and convert them into adjusted *p*‐values based on the Fisher–Stouffer method using the Benjamini–Hochberg procedure (Benjamini & Hochberg, 1995). The LFMM was implemented in the R package LEA (Frichot et al., 2013; Frichot & François, 2015). We used the same environmental variables of the RDA. LFMM uses a Bayesian mixed model approach that employs a PCA to determine the background level of the population structure. The LFMM analysis included population control by incorporating latent factors. The number of latent factors used in LFMM was determined using scree plots, broken stick models, and by examining the dominant axes separating distinct populations in the snmf analysis. We included three latent factors for *D*. *bozinovici*, which has three genetic groups, and four latent factors for *D*. *gliroides*, which has four genetic groups. This strategy ensures that the latent factors appropriately account for the genetic structure observed in each species. To determine loci–environment associations, environmental variables were used as fixed effects, and latent factors were used as random effects (Frichot et al., 2013). We used FDR correction at 5% to export candidate SNPs with adjusted *p*‐values <.05.

### 2.7 Climate adaptation and vulnerability

We used the outlier SNPs found in the genotype–environment association (GEA) analyses to estimate genomic vulnerability. We estimated environmental changes and predicted how climate will change allele frequencies by 2100. We use the R package *AlleleShift* (Kindt, 2021) to model the rate of past evolutionary change and to predict future genomic vulnerability based on the allelic frequency of candidate loci found in RDA and LFMM. An initial two‐step calibration used RDA to build a model and predict the relationship between allele counts and environmental data. Then, predicted allele counts were used as independent variables in a generalized additive model with observed allele frequencies as the response (Blumstein et al., 2020). This step constrains the final allele frequency predictions to fall between 0 and 1. Based on the calibrated allele frequency–environment model, allele counts were predicted for the 2100‐year projected environment taken from the WorldClim database (ssp585, GCM: MIROC6) (Fick & Hijmans, 2017) and then converted to allele frequencies to enable direct comparison. The genomic vulnerability was then expressed as the difference between median values of the observed and predicted allele frequencies between current and projected environmental models (delta allele frequency). To address the relationship between generation length and the rate of allelic change required to keep up with the predicted change, we considered the generation length of *Dromiciops* species used in previous demographic studies (Quintero‐Galvis et al., 2022). The estimated generation length for *Dromiciops* is approximately 3–4 years (Feng et al., 2022; Quintero‐Galvis et al., 2022). Given the rate of environmental change predicted by climate models, we assessed whether the required shifts in allele frequencies are plausible within this timeframe.

### 2.8 Candidate gene identity and function

To examine whether candidate loci harboring outliers SNPs correspond to coding regions, we used the BLASTn algorithm (Zhang et al., 2000) to map reads against the reference genome of *D*. *gliroides* (Accession: PRJNA639670; Feng et al., 2022). For BLASTn runs, we used a maximum e‐value of 10^−5^ to accept/reject an alignment. In the case of mapped loci that correspond to protein‐coding regions, we then examined gene identity and function as summarized by gene ontology terms based on the UniProt database (available at https://www.uniprot.org/uniprot/).

## 3 RESULTS

### 3.1 Genetic diversity

Genomic DNA from 242 individuals were RAD‐sequenced and analyzed, with 1,087,962,937 reads retained after quality filters. Out of these, 191 individuals passed the filters. The average number of reads per sample was 1,856,790. For the dataset of *D*. *bozinovici* with 70 samples 77,131 loci were processed; of these, 24,338 SNPs were variable and kept for further analysis. For the dataset of *D*. *gliroides*, we divided the analysis into two parts. A first analysis with all 121 individuals corresponding to *D*. *gliroides* sensu lato and a second analysis without the subspecies *D*. *gliroides mondaca* (samples of localities 9–12; in other words, *D*. *gliroides* sensu stricto (see D'Elía et al., 2016; Quintero‐Galvis et al., 2022)). For the *D*. *gliroides s*.*l*. dataset 68,469 loci were filtered; of these, 8756 were variable and kept for analyses. In the second analysis, *D*. *gliroides s*.*s*. with 76 samples (localities 13 to 24), 54,653 loci were filtered; of these, 5576 were variable and kept for analyses. Summary statistics for every species, including observed heterozygosity, expected heterozygosity, and F_IS_ are presented in Table 1. Notably, for *D*. *bozinovici*, the specimens sampled at Nahuelbuta (NACH; Figure 1; site in map number 5) showed the highest F_IS_ of 0.30, while the sample from Altos de Lircay (ALCH (1)) showed the lowest F_IS_ of 0.0005. For *D*. *gliroides s*.*l*., the sample from San Martin (SMCH (9)) had the highest F_IS_ of 0.22, and samples from Chiloé Island (CLCH (21) and CDCH (19)) had the lowest F_IS_ values of 0.01. Nucleotide diversity was similar between both species, with a mean *π* of 0.001.

### 3.2 Genetic structure

**FIGURE 2:**
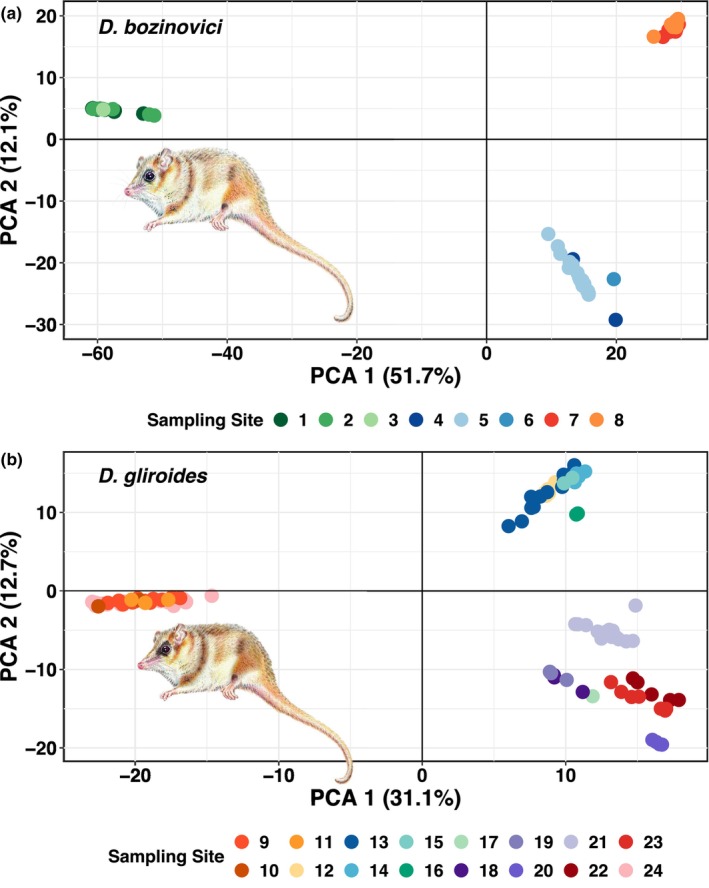
Results of principal component analysis for (a) *Dromiciops bozinovici* and (b) *D*. *gliroides*. The first and second principal components were plotted. The percentage of variance explained by each component is indicated on each axis in brackets. Numbers correspond to sampling localities as indicated in Table 1.

Results from sMNF genetic clustering suggest the most likely number of populations for *D*. *bozinovici* is three (Figure S1a); the same pattern was found in the PCA (Figure 2a). The first cluster (DB‐A) comprises sampling localities 1 to 3 from areas next to the Andes in the northern ranges of *D*. *bozinovici* (Figure 1 and Table 1). The second cluster (DB‐B) consists of samples from the Coastal Cordillera (localities 4 to 6). The third cluster (DB‐C) includes sampling localities 7 and 8 from areas east of the Andes in the southwestern range of *D*. *bozinovici* (Figure 2a). For *D*. *gliroides s*.*l*. the sMNF genetic clustering analysis suggested a K = 4 as the most likely number of clusters (Figure S1b); similarly, the PCA separated the individual genomes of *D*. *gliroides* into four clusters (Figure 2b; Figure S2). Samples from localities 9 to 12 in the Pacific coast of Los Ríos region conform to the first cluster (DG‐A). The second cluster is formed by samples from localities 13 to 17 in the interior area of Los Ríos region (DG‐B). The third cluster (DG‐C) is formed by samples from localities 18 to 21 in southern Pacific coast areas, including Chiloé Island (19–21). The last cluster is composed of samples of localities 22–24, laying in the Argentinean fraction of the species range (DG‐D). The sMNF with 76 samples without cluster DG‐A (*D*. *gliroides mondaca*) suggested a K = 3 as the most likely number of clusters, with a similar number of groups in PCA (Figure S3a,b), corresponding to DG‐B, DG‐C, and DG‐D. *F*_ST_ values between localities ranged from 0.01 to 0.53 for *D*. *bozinovici* and 0.07 to 0.39 for *D*. *gliroides* (Tables S1 and S2).

Results from the hierarchical AMOVA comparing the three clusters of *D*. *bozinovici* showed a significant genetic differentiation among them, with 68.3% of the observed molecular variance explained by differences among clusters (F_CT_ = 0.70), 14.1% explained by differences among populations within clusters (F_SC_ = 0.44), and 20.2% explained by differences within populations (*F*_ST_ = 0.44) (Table S3). The AMOVA comparing the four clusters of *D*. *gliroides* showed that 55.6% of the observed molecular variance is due to differences among clusters (F_CT_ = 0.50), 24.1% explained by differences among populations within clusters (F_SC_ = 0.34), and 20.2% explained by differences within populations (*F*_ST_ = 0.36) (Table S3).

### 3.3 Isolation by distance and isolation by environment

**FIGURE 3:**
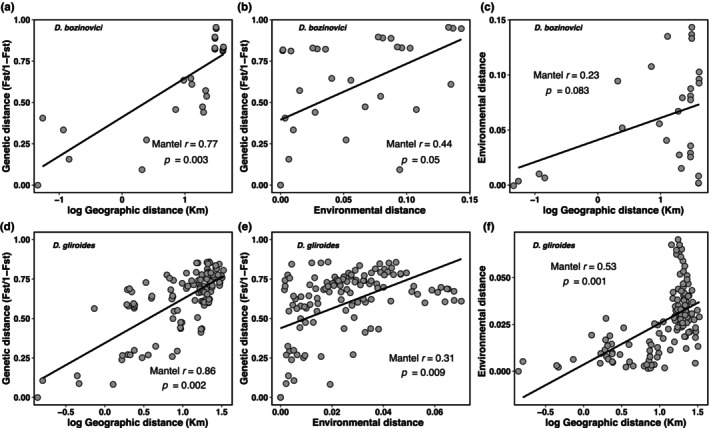
Isolation by distance and isolation by environment, and correlation of environmental distance with the logarithm geographic distance of (a–c) *Dromiciops bozinovici* and (d–f) *D*. *gliroides*.

There was a strong and significant correlation between genetic and geographic distances for both species (*D*. *bozinovici*: Mantel *r* = .77, *p* = .003, Figure 3a; *D*. *gliroides*: Mantel *r* = .86, *p* = .002, Figure 3d), as the genetic distance between population pairs increases linearly with geographic distance, indicating a significant pattern of IBD. We also identified a significant pattern of IBE based on distance derived from environmental deviation for *D*. *gliroides* (Mantel *r* = .31, *p* = .009; Figure 3e), but not for *D*. *bozinovici* (Mantel *r* = .44, *p* = .05; Figure 3b). Likewise, the autocorrelation between environmental and geographic distances was strong for *D*. *gliroides* (Mantel *r* = .53, *p* = .001; Figure 3f) but not significant for *D*. *bozinovici* (Mantel *r* = .23, *p* = .08; Figure 3c).

### 3.4 Outlier detection

The number of putative loci under selection (outlier loci) found varied among the three methods employed. For *D*. *bozinovici*, the number of detected outlier loci was 32 with Bayescan, 6857 with fdist2, and 8336 with *pcadapt*. Only six loci were common among the three methods (Figures S3a, S4, and S5). Of these six loci, one candidate locus (309652) was mapped to one section of a known protein‐coding gene in the reference genome; it corresponds to the MEF2D gene. The other five loci were mapped to non‐coding regions from chromosomes 3 and 4 (Table S4). Similarly, the number of putative loci under selection identified for *D*. *gliroides* varied among the three methods. Bayescan identified 305, fdist2 identified 602, and *pcadapt* identified 1243 outliers (Figures S6a and S7). Although we based analysis on a single SNP per RADtag/locus, we subsequently examined additional SNPs on the RADtags to determine if they exhibited similar patterns of variation. This follow‐up analysis helps to discern whether the observed patterns are due in fact to selection and not to genetic drift, providing further validation of our results. Forty‐three outlier loci were common for all three methods. Of these 43 loci, 17 were mapped in different non‐coding regions. The other 26 loci were mapped to different sections of known protein‐coding genes that mediate several biological processes, including skeletal and muscular functions (Table S5). The allelic frequency of the 43 loci at the different *D*. *gliroides s*.*l*. sites varied, with some loci showing lower allelic frequency in cluster DG‐A (Figure 1; localities 9–12) compared to other clusters. In some cases, one of the alleles at these loci appeared to be near fixation or lost within DG‐A, suggesting reduced genetic variation at these sites (Figure S6). The patterns of loci in linkage with these outlier SNPs showed similar allele frequency patterns, suggesting that selective sweeps may be influencing these regions. The number of putative loci under selection identified for *D*. *gliroides s*.*s*. (i.e., without samples of cluster DG‐A) identify only two outlier loci for three methods, and both were mapped to different sections of known protein‐coding genes (Table S6; Figures S8a and S9).

To visualize if there were differences in the candidate loci among the genetic clusters found for each species, we repeated the analyses focused on every genetic cluster of each species. The results revealed no common putative loci across all three methods for any cluster of both species (Figures S4b–S6b).

### 3.5 Genotype–environment association

**FIGURE 4:**
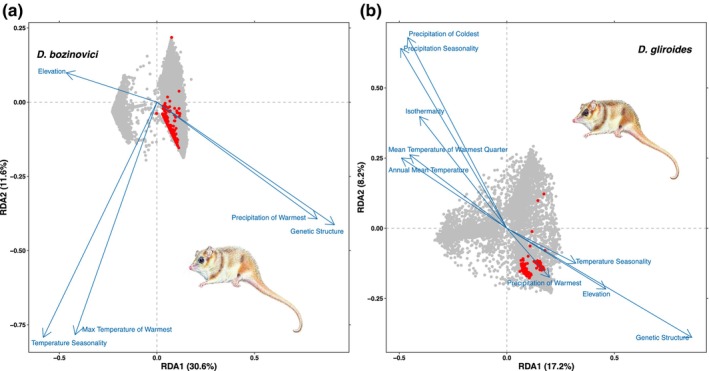
Redundancy analysis (RDA) using environment variables from WorldClim and elevation for (a) *Dromiciops bozinovici* and (b) *D*. *gliroides*. The direction of the main variation for each constraining variable is indicated by a blue vector in *D*. *bozinovici* and *D*. *gliroides*. The blue vectors represent the direction of variation for the relevant environmental variables and genetic structure, the gray points are SNPs, and the red points represent putative SNPs of selection most strongly correlated with variables.

The RDA for *D*. *bozinovici* identified 436 loci with frequencies significantly correlated to environmental variation (*F* = 9.12, *p* = .001). A biplot of RDA1 versus RDA2 showed that samples were distributed into three groups (Figure S12a; Figure 4a). The percentage of the variation attributed to population structure was 4.67%. The proportion of the variance explained by the environmental predictors was 44.1% (*R* ^2^ = .46). The results of the RDA indicate that the first eigenvalue explained 30.6% of the total genetic variance, the second eigenvalue explained 11.6%, and the third eigenvalue 1%. The high values of elevation, the maximum temperature of the warmest month (bio 5), and temperature seasonality (bio 15) were associated with samples of the north of the Andes (DB‐A group, localities 1–3). On the other hand, precipitation of the warmest quarter (bio 18) was associated with samples from east of the Andes in the southwestern DB‐C group localities (localities 7–8). Interestingly, most candidate loci for selection were associated with precipitation of the warmest quarter (Figure S12b; Figure 4b). Results of the LFMM analysis for *D*. *bozinovici* identified seven putative outlier loci (*p* < .01). Seven loci were common between LFMM and RDA. Three of the seven loci were mapped to the section of a known protein‐coding gene in the reference genome (Table S7). The allelic frequency of the three putative loci mapped to the genome did not present changes among localities (Figure S9). No common loci were found between the five methods (BayeScan, *pcadapt*, fdist2, LFMM, and RDA) for *D*. *bozinovici*.

Results from the RDA for *D*. *gliroides* indicate that the pattern of genetic variation correlates with environmental variation (*F* = 6.54, *p* = .001), identifying 201 putative outlier loci (Figure 4b). RDA1 and RDA2 divided the samples into four clusters (Figure S12b). The percentage of the variation attributed to population structure was 6.8%. The proportion of the variance explained by the environmental predictors was ca. 42% of the total variation (*R* ^2^ = .34). The RDA explained 17.2% of the genetic clustering variance with the first eigenvalue, 8.2% with the second eigenvalue, and 1.3% with the third eigenvalue. The four localities of cluster DG‐A (localities 9–12) were associated with high values of the mean temperature of the warmest quarter, annual mean temperature, and isothermality. The variables precipitation of the warmest quarter, elevation, and temperature seasonality were associated with samples from the southern portion of the Coastal Cordillera, including Chiloé Island (DG‐C; localities 18–21) and Argentinean samples (DG‐D; localities 22–24). The RDA for *D*. *gliroides s*.*s*. (i.e., without samples of cluster DG‐A) explained 15.7% of the genetic clustering variance with the first eigenvalue and 6.4% with the second eigenvalue (Figure S13). The pattern of genetic variation was similar to that found in the RDA with all *D*. *gliroides s*.*s*. samples. Most of the candidate loci under selection were associated with the variable precipitation of the warmest, precipitation seasonality, annual mean temperature, and mean temperature of the warmest quarter (Figure S13b; Table S9). Results of the LFMM analysis for *D*. *gliroides* identified four putative outlier loci (*p* < .01). Four loci were common between the two methods (RDA and LFMM). Two candidate loci were mapped to coding regions (ALG8 and PRKN), and two were mapped to an uncharacterized protein (Table S6, Figure S11). Combining the candidate loci for selection among the five methods for *D*. *gliroides*, one common locus that falls on chromosome 3 was found (Locus 663,418; Tables S5–S8) corresponding to gene ALG8 (alpha‐1,3‐glucosyltransferase). The function of this protein relates to transferring glucose from dolichyl phosphate glucose (Dol‐P‐Glc) onto the lipid‐linked oligosaccharide Glc1Man9GlcNAc2‐PP‐Dol before it is transferred to the nascent peptide, required for PKD1/Polycystin‐1 maturation and localization to the plasma membrane of the primary cilia (Wu et al., 2022).

### 3.6 Climate adaptation and vulnerability

**FIGURE 5:**
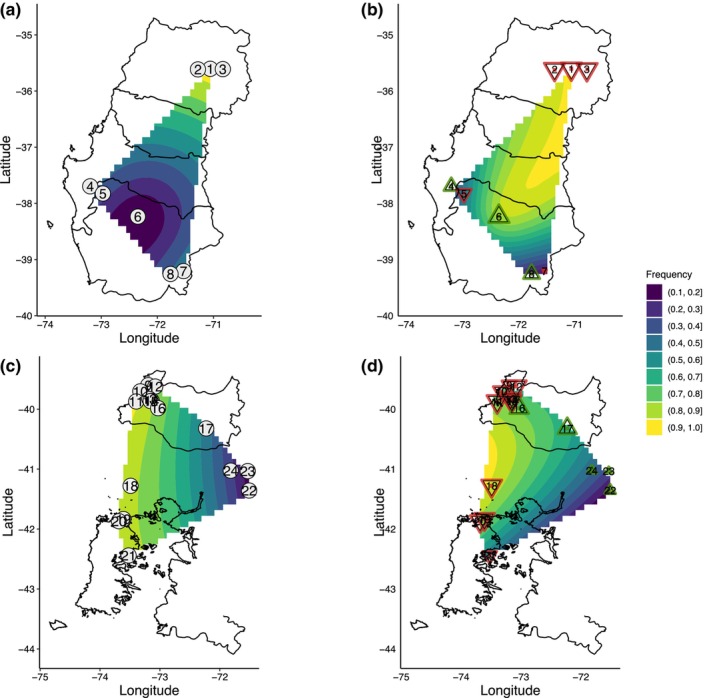
Depiction of changes in allele frequencies of candidate loci identified by genome–‐environment association analyses via AlleleShift candidate. (a) Frequencies in the present climate for the locus 386,373 for *D*. *bozinovici*. (b) Frequencies inferred for the same locus in the future (year 2100) for *D*. *bozinovici*. (c) Frequencies in the present climate for the locus 663,418 for *D*. *gliroides*. (d) Frequencies inferred for the same locus in the future (year 2100) for *D*. *gliroides*. Gray circles with numbers correspond to sampling localities (see Table 1). Changes in frequencies are color‐coded: the red triangle means decreasing and the green triangle means increasing.

We modeled the rate of past evolutionary change and predicted future genomic vulnerability based on the allelic frequency of candidate loci found in association with environmental variation through GEA analyses. Genetic structure was a reasonable predictor of genomic vulnerability to *D*. *bozinovici* (*R* ^2^ = .90, *p* < .001) and *D*. *gliroides* (*R* ^2^ = .92, *p* < .001). The seven loci under selection in the eight *D*. *bozinovici* localities do not show a large change in allele frequency projected into the future, except for samples from localities in the southern part of the species distribution (localities 7 and 8; Figures S15 and S16). The locus 386,373, mapped to the PTGES3 gene, shows a decline in projected allelic frequency in the northern distribution and an increase in the southern localities of *D*. *bozinovici* (Figure 5a,b).

The allelic frequency of the four loci under selection in the 16 localities of *D*. *gliroides s*.*l*. showed a distinct pattern. Locus 1,017,511 showed a decrease in the projected allelic frequency in almost all localities in the future. In contrast, locus 847,298 showed an increase in its allele frequency for the samples of the DG‐C group (localities 18–21) near Chiloé Island (Figures S17 and S18). Locus 663,418 mapped to gene ALG8 and shows an increase in its allele frequency projected into the future in Andean localities (17, 22–24) and a decrease in allele frequency in the future in localities in the Valdivian coast corresponding to the group DG‐A (localities 9–10) (Figure 5c,d).

## 4 DISCUSSION

Our work addresses relevant questions about the relative importance of genome‐wide levels of genetic variation and the association of environmental variables with allelic frequencies of thousands of SNPs in two Valdivian Forest endemic marsupial species of the genus *Dromiciops*. Notably, our analysis shows the spatial correlation between allelic frequencies and environmental variables such as precipitation and temperature in both *D*. *bozinovici* and *D*. *gliroides*. These loci may suggest local adaptation as a result of a process of specialization to specific habitats (Wadgymar et al., 2022). Our models suggest that the predicted shifts in allele frequencies are within the range of plausible evolutionary responses given these species' generation time and historical rates of evolutionary change (Feng et al., 2022; Quintero‐Galvis et al., 2022). However, rapid environmental changes may still pose a significant challenge, highlighting the need for conservation efforts to mitigate potential genomic vulnerabilities. Vazquez et al. (2022) showed that precipitation and several temperature‐related variables greatly influence the distributional range of both species of *Dromiciops*. Our findings, coupled with previously delimited suitability zones, allow the identification of outlier allelic frequency differences consistent with patterns of adaptation to specific environmental areas for both species of *Dromiciops*.

Our investigation sheds light on the crucial interplay between genetic variation, environmental factors, and the potential adaptation of *Dromiciops* to distinct habitats. These findings underscore the significance of understanding how specific environmental variables may influence genetic structure, aiding in the identification of vulnerable areas crucial for the conservation of these species. For instance, populations in the northernmost range (e.g., those found in localities 1–3 (cluster DB‐A) of *D*. *bozinovici*) exhibit unique genetic variants that may be of relevance for adapting to specific climatic conditions. Additionally, populations in cluster DG‐A (localities 9–12) of *D*. *gliroides* showed lower allelic diversity and possible signs of reduced genetic variability, indicating these areas might require focused conservation efforts to maintain genetic diversity. These areas should be prioritized for conservation as they harbor unique genetic variants likely crucial for the species' long‐term adaptability and survival. Specifically, conserving these populations can help preserve the genetic diversity necessary for the species to adapt to ongoing and future environmental changes. Given the strong association between distinct aspects of the natural history of *Dromiciops* with forests and their seasonal changes (Fontúrbel et al., 2022; Nespolo, Sáenz‐Agudelo, et al., 2022), identifying vulnerable areas within the range of *Dromiciops* will significantly contribute to the design of biologically sound conservation strategies (Nespolo et al., 2024).

### 4.1 Population structure and significant variables

By enlarging the sampling used by Quintero‐Galvis et al. (2022), here we show that *D*. *bozinovici* showed a pronounced genetic structure with three highly differentiated genetic clusters. Among these, the distinction of the populations corresponding to northeastern cluster DB‐A (localities 1–3; Table 1; Figure 1) is remarkable as differentiation between the other two groups, DB‐B and DB‐C, was comparatively more subtle (Table 1; Figure 2; Figure S1). The localities of cluster DB‐A were situated next to the Andes in the northern ranges of *D*. *bozinovici*. These findings expand those of a previous study based on mitochondrial and nuclear genes that indicated substantial genetic structuring and differentiation within *D*. *bozinovici* (Quintero‐Galvis et al., 2021). Our results suggest that the observed genomic structure in *D*. *bozinovici* results from gene flow restrictions as a function of geographic distances among sampled localities. We detected a significant IBD pattern, indicating that geographic distance plays a crucial role in shaping genetic structure. However, the correlation of genetic and environmental distances was not significant, nor was the comparison between environmental and geographic distances. This suggests that environmental differences alone appear to have a minimal influence on the observed genetic patterns. While there is some correlation between geographic and environmental distances, our data indicate that geographic distance is the primary driver of the genomic structure observed. Nonetheless, the complexities of gene flow, barriers to gene flow, and genetic drift must also be considered in understanding the full picture of population structure (Pyron et al., 2023).

The RDA supports the hypothesis initially stated in the introduction that *D*. *bozinovici* exhibits a small degree of ecological adaptation across its range. Specifically, the allelic frequencies of clade DB‐A (localities 1–3) were correlated with precipitation, temperature changes, and elevation. These localities do not show significant changes in future projection models, except for locus 386,024 (Figure S13). In contrast, in the southern part of the species range (localities 6–8), locus 386,373 shows an increase in allelic frequency in future models (Figure 5b). Of note, temperature and seasonal precipitation variables were correlated with allelic frequencies in southern populations of *D*. *bozinovici*. Moreover, the results of the vulnerability analysis indicate that the allelic frequencies of loci under selection in cluster DB‐A (localities 1–3) are expected to remain relatively stable. This stability is crucial for conservation management because *D*. *bozinovici* inhabits forests that are highly impacted by human activities, which could otherwise exacerbate genetic vulnerabilities (Luebert & Pliscoff, 2017; Vazquez et al., 2022). By understanding how specific alleles respond to environmental changes, conservation strategies can be tailored to protect these genetically distinct populations. For instance, preserving areas that maintain current climatic conditions could be vital for the stability of these allelic frequencies in this species.

The southern species *D*. *gliroides s*.*l* is also highly structured; it shows four genetic groups. While Quintero‐Galvis et al. (2022), based on SNPs, reported that *D*. *gliroides* was structured into three genetic groups, the inclusion here of additional populations uncovered another genetic group, which was previously found in nuclear and mitochondrial DNA analysis (Himes et al., 2008; Quintero‐Galvis et al., 2021). Our result indicates that *D*. *gliroides mondaca* (DG‐A; localities 9–12) is the most genetically divergent group; in fact, D'Elía et al. (2016, 2020) consider this group as a distinct species, while Quintero‐Galvis et al., 2022 consider it a subspecies (*D*.*g mondaca*). The DG‐A cluster is geographically closer to the DG‐B cluster but separated by the Cruces River. The area inhabited by DG‐A holds several endemic species and has been reported as a glacial refuge dating back to the middle Miocene (Malumián & Náñez, 2011; Montemayor et al., 2017; Nuñez et al., 2011; Rabassa et al., 2011).

Samples of *D*. *gliroides gliroides*, without those of *D*. *gliroides mondaca*, also show high structure in three distinct genetic groups. These are the groups DG‐B (localities 13–17), DG‐C, corresponding to southern Chilean localities, including Chiloé Island (localities 18–21) and DG‐D for southern Argentinean localities (22–23). The pronounced genetic structure observed in Chiloé Island, together with the sample from locality 18 may be attributed to significant genetic drift caused by the small population size of the ancestral population shortly after divergence (see Quintero‐Galvis et al., 2022). During the Last Glacial Maximum, the island of Chiloé was not isolated from the continent and its south‐eastern half was covered by ice (Hulton et al., 2002; Lessa et al., 2010; McCulloch et al., 2000); as such its north‐western portion of the island of Chiloé and the coastal mainland have been suggested as a glacial refugium (Hulton et al., 2002; Moreno et al., 1994; Rabassa, 2008; Villagrán & Hinojosa, 1997). Our findings align with existing evidence indicating the persistence of a land connection between Chiloé Island and the continent during Pleistocene glacial oscillations, owing to a substantial drop in sea level (D'Elía et al., 2013, 2015; Heusser et al., 1996; McCulloch et al., 2000; Palma et al., 2005; Valdez & D'Elía, 2018). Testing for IBD and IBE in *D*. *gliroides* indicated that geographic and environmental distances were equally important in explaining the observed genetic differences. Indeed, the relationship between geographic and environmental distances was highly correlated, which made it difficult to correctly parse out the factor that plays the key role in shaping the species' genetic variation.

According to the RDA results, environmental variables explain 42% and 44% of the observed genetic variation in *D*. *gliroides* and *D*. *bozinovici*, respectively. Among these variables, precipitation during the warmest quarter of the year (bio 18) was a significant variable in common between both species (Fick & Hijmans, 2017). Recent studies have indicated that precipitation and temperature represent the main environmental determinants of the distribution of the two species of *Dromiciops* (Vazquez et al., 2022). Furthermore, meta‐analyses have demonstrated that local precipitation is a significant selective force in wild populations of invertebrates, plants, and vertebrates (Myers‐Smith & Myers, 2018; Siepielski et al., 2017, 2018), including species partially sympatric with the species of *Dromiciops* (Giorello et al., 2018; Teta et al., 2022). Our results suggest that local precipitation (Precipitation of the warmest quarter: bio 18) and temperature (Temperature seasonality: bio 4 and Annual mean temperature: bio 1; Mean temperature of the warmest quarter: bio10) are potentially important selective forces for *Dromiciops*. It is also crucial to consider that climate change may cause widespread changes in selection regimes for *Dromiciops* species. The impact of these environmental variables could be mediated by the physiological responses of these marsupials to extreme temperature fluctuations and limited food availability (Cooper et al., 2016; Giroud et al., 2021; Nespolo et al., 2021, 2024; Withers et al., 2006). *Dromiciops* face harsh winter environmental conditions with torpor, which involves changes in cellular metabolism, including specific changes to gene expression that are coordinated by different genes and by the posttranscriptional gene silencing activity of microRNAs (miRNA) (Breedon et al., 2023; Hadj‐Moussa et al., 2016; Luu et al., 2018; Wijenayake et al., 2018). Nevertheless, the genes identified here are not those found in previous studies on the response of *Dromiciops* to torpor and temperature changes (Nespolo et al., 2018). This discrepancy might be due to the limitations of Rad‐seq, which samples a small fraction of the genome (<<1%), potentially overlooking many loci of interest and lacking the power to detect weakly selected loci (Maier et al., 2023). To verify whether we have sequence data from those genes, we conducted a BLAST/BEDtools analysis. The results indicated that while we have some coverage of regions associated with torpor and hibernation, these genes did not appear as outliers in our dataset. Despite these limitations, our study inferred a clear correlation between precipitation and temperature variables and the adaptive response of *Dromiciops*. Future research could benefit from utilizing more comprehensive genomic approaches, such as whole‐genome sequencing or targeted gene expression studies, to identify genes specifically associated with molecular functions involved in torpor and other adaptive responses. Such approaches would provide a deeper understanding of the genetic basis of adaptation to environmental changes in *Dromiciops*, facilitating more effective conservation strategies.

Fruits of several plant species are important in the diet of *Dromiciops* (Fontúrbel et al., 2022), especially posterior to hibernation time (Nespolo, Fontúrbel, et al., 2022). The flowering and fruiting times for fleshy fruits are strongly conditioned by precipitation and temperature (Amico & Aizen, 2000; Amico et al., 2009; Fontúrbel et al., 2022). A study of over 7 years showed that populations of *D*. *gliroides* are affected by droughts, reducing their abundance by half (Balazote Oliver et al., 2017). Thus, precipitation should strongly impact the fitness of specimens of *Dromiciops*. Additionally, temperature variation influences the activity patterns of *Dromiciops*, making individuals more active during nights with constant temperatures (Fontúrbel et al., 2014, 2022). As such, allelic frequency differences in outlier loci associated with precipitation and temperature may be indicative of adaptive responses to habitat differences. A special case is found when allelic frequencies of GEA outliers of Andean *D*. *gliroides* and *D*. *bozinovici* are projected to the future, where we observed the importance of local adaptation to changes in precipitation and temperature. Although it is premature to conclude that *Dromiciops* populations are locally adapted before a direct causal relationship between genotype, phenotype, and effect on fitness is established, the identification of a correlation between allele frequency and environmental variables provides indirect evidence for natural selection, warranting future study (Lemay & Russello, 2014). Additional research is necessary to identify the traits that could be under selection.

Genomic signatures provide a possible indication of climate adaptation by *Dromiciops*. This is relevant as global warming studies suggest that temperature will increase in the Valdivian Forests while precipitation will be reduced in some areas (Lippmann et al., 2019). Consequently, specimens of *Dromiciops* are expected to face increased drought and heat stress. These conditions could pose significant challenges to their survival and reproductive success, emphasizing the need for conservation strategies that consider these environmental changes (Nespolo et al., 2024). The Chilean Valdivian Forest hotspot is of maximum priority for conservation due to its high species richness and endemicity in an ecosystem facing much human‐induced degradation. Climate change is one of the severe pressures on the viability of many species (Fuentes‐Castillo et al., 2020). In fact, studies evaluating the role of *Dromiciops* as a disperser of the plant mistletoe suggest a cascade of effects due to climate change (Fontúrbel et al., 2018). Therefore, it is crucial to emphasize that changes in temperature and precipitation will generate essential changes in *Dromiciops* populations. These changes could have significant downstream effects on ecosystems, particularly on plant species that rely on *Dromiciops* for seed dispersal (Amico & Aizen, 2000; Fontúrbel & Medel, 2017). As such, decreases in populations of *Dromiciops* may lead to altered plant community dynamics, affecting biodiversity and ecosystem stability (Fontúrbel et al., 2022). Acknowledging these interconnected relationships underscores the importance of conserving *Dromiciops* and mitigating the impacts of climate change on their populations.

### 4.2 Adaptative loci and function

The assessment of candidate loci for selection revealed differences between the two species. *D*. *gliroides* had the greatest number of candidate loci under selection. However, only locus 663,418, consistently identified using all five outlier detection methods, mapped to one coding region, the gene ALG8 (alpha‐1,3‐glucosyltransferase) located on chromosome 3. While the exact function of alpha‐1,3‐glucosyltransferase can vary depending on the cell type and biological context, its generally associated with synthesizing specific carbohydrates in cell membranes and modifying glycoproteins and glycolipids (Apple et al., 2023). Although this gene has been linked to cancer in other species (Wu et al., 2022), its role in *Dromiciops* may involve other processes. The frequency of one of the alleles of locus 663,418 remained high across all *D*. *gliroides* localities in the present time, except for those in the DG‐D cluster, where it displayed a low frequency (Figure S7). For DG‐D (localities of Chiloé Island), understanding the specific genetic markers and their environmental correlations is crucial for targeted conservation strategies. This knowledge can guide efforts to monitor and preserve genetic diversity within DG‐D populations, particularly as they may face unique environmental pressures. Effective management practices should consider these genetic factors to ensure the resilience of DG‐D populations in the face of climate change and habitat alterations. These shifts in adaptive allele frequencies underscore a high genomic vulnerability to climate change, potentially impacting fitness. While we used a conservative approach, applying five different methods to detect outliers, these methods may still face limitations by each method. It is important to note that these loci are identified as outliers with respect to *F*_ST_ and may or may not be directly related to local adaptation, especially considering the potential influence of genetic drift (Hohenlohe et al., 2021; Narum & Hess, 2011; Rellstab et al., 2015; Thörn et al., 2021). Therefore, findings related to any one locus should be interpreted with caution, acknowledging the potential limitations and need for further validation.

*Dromiciops bozinovici* exhibited fewer outlier loci, with only one candidate locus mapped to a known protein‐coding region. Locus 309,652, mapped on chromosome 4, matched gene MEF2D (Table S4), which is involved in diverse regulatory functions, including control over cell growth, survival, and apoptosis via the p38 MAPK signaling pathway, regulating neuronal apoptosis, particularly in muscle‐specific and growth factor‐related transcription (Mohawk et al., 2019). The GEA analysis for *D*. *bozinovici* found seven outlier loci associated with environment variables, of which only three had well‐defined functions (Table S7). Locus 119,347 mapped to gen FES, a tyrosine‐protein kinase responsible for downstream signaling from cell surface receptors, which regulates essential cellular functions, including the actin cytoskeleton, microtubule assembly, cell attachment, and cell spreading (Chung et al., 2014). Locus 386,373, mapped to the gene PTGES3; this cytosolic prostaglandin synthase that catalyzes the conversion of prostaglandin endoperoxide H2 (PGH2) to prostaglandin E2 (PGE2) (Table S7), significant expression in normal birth processes in marsupials (Renfree et al., 1994; Waclawik et al., 2006). The allelic frequency of these loci showed interesting patterns, particularly in localities 1–3 of *D*. *bozinovici*, projecting a decline in allelic frequency linked to precipitation as the primary variable. This indicates a significant predictive relationship between this environmental factor and genomic vulnerability amid global warming. While the decline in allelic frequency linked to temperature or precipitation could indicate genomic vulnerability to global warming, it does not necessarily mean that the localities will become uninhabitable (Hoffmann et al., 2021). However, it does raise concerns about the potential impact of climate change on the genetic diversity and resilience of the populations in those areas (Blumstein et al., 2020; Lotterhos, 2024). We recognize the limitations of extrapolating findings from a single locus to broader conclusions about habitat viability or population vulnerability is essential.

The identified genes and their potential roles in physiological stress responses, such as those linked to the MAPK signaling pathways and prostaglandin synthesis, suggest that *Dromiciops* populations may face increased challenges under climate change scenarios. This has significant implications for conservation and management, highlighting the need to consider these genetic factors in developing strategies to mitigate the impacts of climate change on these species. Although this study primarily focuses on delineating the pattern of outlier loci across the species distributional ranges, our interpretation is based on allelic frequencies correlation to environmental variables. While we cannot define if the functions of the genes mentioned in this study directly affect the performance of specimens of *Dromiciops*, this is the first report of candidate genes related to the adaptation of natural populations to different environments in this genus of highly divergent South American marsupials, and largely for any South American marsupial. Future studies could elucidate the precise roles of the identified genes and their implications for the long‐term viability of populations of *Dromiciops*. Furthermore, exploring how these genes interact with environmental variables could provide valuable insights into the broader context of local adaptation and population dynamics. Our findings provide a robust foundation to explore further the possible environmental forces driving genetic differentiation and local adaptation of the population of *Dromiciops*, which in turn could allow the development of biologically sound conservation strategies for these species.

## 5 CONCLUSION

The present study represents the first study combining population and landscape genomic approaches to identify candidate loci under positive selection in New World marsupials; the study gains relevance as the studied species are likely threatened by climate change. Genomic analyses have revealed a high degree of population structure among populations of *D*. *bozinovici*. Additionally, these results corroborate and expand an already known pattern of genetic structuring in *D*. *gliroides*. These patterns of population structuring may be associated with various factors such as geography and the geologic and climatic history of the areas. Furthermore, our study shows that variation patterns of some genes, which differ from the pattern of the whole genome, are associated with environmental differences and likely local adaptation. These genomic findings provide a broader perspective on the evolution and population dynamics of both species of *Dromiciops*. Moreover, these findings suggest that the observed genetic structure could significantly impact conservation efforts, emphasizing the necessity to consider these genetic subdivisions when envisioning conservation strategies tailored to the particularities of each population cluster. Expanding on how these genetic insights can inform management practices is crucial, given the strong conservation implications. Current conservation efforts might need to be adjusted to account for the genetic diversity and structure within *Dromiciops* populations. Specifically, identifying population clusters that harbor unique genetic variation is essential for prioritizing areas for protection. For instance, conservation strategies could focus on preserving areas that maintain current climatic conditions to support the stability of adaptive alleles.

Our study shows the relevance of employing diverse approaches in landscape genomic analysis to identify local adaptation. Recognizing the susceptibility of these algorithms to false positives, we followed a conservative approach using multiple methodologies to detect outliers and kept only those loci single out by all methods. The primary environmental variable associated with local adaptation in both species of *Dromiciops* was the warmest quarter precipitation and secondarily temperature seasonally and elevation variables, suggesting avenues for further exploration through models integrating genomic data and Species Distribution Models, which would hold immense promise as pivotal indicators for forecasting the responses of the species of *Dromiciops* to climate fluctuations. Furthermore, these findings lay the foundation for innovative strategies aimed at preserving wildlife in changing environments.

## AUTHOR CONTRIBUTIONS

**Julian F. Quintero‐Galvis:** Conceptualization (equal); data curation (lead); formal analysis (lead); funding acquisition (equal); investigation (equal); methodology (equal); project administration (equal); resources (equal); software (equal); writing – original draft (equal); writing – review and editing (equal). **Pablo Saenz‐Agudelo:** Conceptualization (equal); data curation (equal); methodology (equal); supervision (equal); validation (equal); writing – original draft (equal); writing – review and editing (equal). **Guillermo D'Elía:** Conceptualization (equal); investigation (equal); project administration (equal); supervision (equal); validation (equal); writing – original draft (equal); writing – review and editing (equal). **Roberto F. Nespolo:** Conceptualization (equal); funding acquisition (equal); investigation (equal); project administration (equal); supervision (equal); validation (equal); writing – original draft (equal); writing – review and editing (equal).

## CONFLICT OF INTEREST STATEMENT

The authors declare no competing interests.

## Supporting information


Table S1.–S9.

Figure S1.–S18.

## Data Availability

Data associated with this study have been deposited in the National Center for Biotechnology Information (NCBI) Sequence Read Archive under the BioProject accession number PRJNA1088453; SRA: SUB14315196.
