# Supplementary material for: Local adaptation of Dromiciops marsupials (Microbiotheriidae) from southern South America: Implications for species management facing climate change
Source: Ecol Evol. 2024 Oct 3;14(10):e70355. doi: 10.1002/ece3.70355 (PMC11450259; doi:10.1002/ece3.70355)
Supplement: Supplementary file 2 — Figure S1.–S18. [file ECE3-14-e70355-s002.pdf]

JOURNAL: ECOLOGY AND EVOLUTION

SUPPORTING INFORMATION

Local adaptation of *Dromiciops* marsupials (Microbiotheriidae) from southern South America: implications for species management facing climate change

Julian F. Quintero-Galvis[1,2,*], Pablo Saenz-Agudelo[1,3], Guillermo D'Elía[1,4] & Roberto F. Nespolo[1,2,5,6]

[1.] *Instituto de Ciencias Ambientales y Evolutivas, Universidad Austral de Chile, Valdivia. Chile.*

[2.] *Millennium Nucleus of Patagonian Limit of Life (LiLi), Valdivia, Chile.*

[3.] *Millenium Nucleus for ecology and conservation of temperate mesophotic reefs (NUTME), Las Cruces, Chile.*

[4.] *Colección de Mamíferos, Universidad Austral de Chile, Valdivia, Chile.*

[5.] *Center of Applied Ecology and Sustainability (CAPES), Facultad de Ciencias Biológicas, Universidad Católica de Chile, Santiago 6513677, Chile.*

[6.] *Millennium Institute for Integrative Biology (iBio), Santiago, Chile.*

***Corresponding author**

E-mail address: julianquintero1924@gmail.com (Julian F. Quintero-Galvis)

Table list

Table S1. Pairwise F_{ST} values based on Weir and Cockerham's unbiased genetic distances (1984) for all localities estimated from 2,640 SNPs for *Dromiciops bozinovici*. The initials of the localities correspond to the names in the table1.

Table S2. Pairwise F_{ST} values based on Weir and Cockerham's unbiased genetic distances (1984) for all localities estimated from 2,640 SNPs for *Dromiciops bozinovici*. The initials of the localities correspond to the names in the table1.

Table S3. Analysis of Molecular Variance (AMOVA) for *Dromiciops bozinovici* comparing three genetic clusters and *D. gliroides* comparing four genetic clusters

Table S4. Loci identified as putatively under selection by three methods (BayeScan, pcadapt, and fdist2) for *Dromiciops bozinovici.*

Table S5. Loci identified as putatively under selection by three methods (BayeScan, pcadapt, and fdist2) for *Dromiciops gliroides.*

Table S6. Loci identified as putatively under selection by three methods (BayeScan, pcadapt, and fdist2) for *Dromiciops gliroides* without clade DG-A.

Table S7. Putative candidate loci commonly identified by genome–environment association analyses using LFMM and RDA for *Dromiciops bozinovici.*

Table S8. Putative candidate loci commonly identified by genome–environment association analyses using LFMM and RDA for *Dromiciops gliroides.*

Table S9. Putative candidate loci commonly identified by genome–environment association analyses using LFMM and RDA for *Dromiciops gliroides* without cluster DG-A.

Figure List

Figure S1. Barplot of individual ancestry proportions inferred with sNMF for **a-b)** *Dromiciops bozinovici* **c-d)** *Dromiciops gliroides*. Codes in the top correspond to sampling sites in Table 1. Panels on the left show the cross-entropy values as a function of K (1–10). K = 3 for *Dromiciops bozinovici* and K = 4 for *Dromiciops gliroides* were the best value of cross entropy.

Figure S2. Results of principal component analysis for a) *Dromiciops bozinovici* and b*) D. gliroides.* The first and third principal components were plotted. The percentage of variance explained by each component is indicated on each axis in brackets. Numbers correspond to sampling localities as indicated in Table 1.

Figure S3. Barplot of individual ancestry proportions inferred with sNMF for **a-b)** *Dromiciops gliroides* (Localities 13 to 24). Codes in the top correspond to sampling sites in Table 1.

Figure S4. a) Venn diagram showing the number of loci identified as putatively under selection for *D. bozinovici* by each method (BayeScan, pcadapt, and fdist2). **b)** Venn diagram showing the number of loci identified as putatively under selection between genetics clusters of *D. bozinovici* using three methods. Figure indicates the exclusives loci detected of each method and the common ones between two and three methods.

Figure S5. Major allele frequency for putative candidate loci identified by three methods (BayeScan, *pcadapt,* and fdist2) for *Dromiciops bozinovici.* The function of the gene associated with the locus is in Table S4. The locus name corresponds to the ID locus in Table S4

Figure S6. a) Venn diagram showing the number of loci identified as putatively under selection for *D. gliroides* using three methods (BayeScan, pcadapt, and fdist2). **b)** Venn diagram showing the number of loci identified as putatively under selection between genetics clusters of *D. gliroides* using three methods. Figure indicates the exclusives loci detected of each method and the common ones between two and three methods.

Figure S7. Major allele frequency for putative candidate loci identified by three methods (BayeScan, *pcadapt,* and fdist2) for *Dromiciops gliroides.* The function gene associated with the locus is in Table S5. The locus name corresponds to the ID locus in Table S5.

Figure S8 a) Venn diagram showing the number of loci identified as putatively under selection for *D. gliroides* without clade DG-A using three methods (BayeScan, pcadapt, and fdist2). **b)** Venn diagram showing the number of loci identified as putatively under selection between genetics clusters of *D. gliroides* using three methods. Figure indicates the exclusives loci detected of each method and the common ones between two and three methods.

Figure S9. Major allele frequency for putative candidate loci identified by three methods (BayeScan, *pcadapt,* and fdist2) for *Dromiciops gliroides* without cluster DG-A. The function gene associated with the locus is in Table S6. The locus name corresponds to the ID locus in Table S6.

Figure S10. Major allele frequency for putative candidate loci identified by genome–environment association analyses using LFMM and RDA for *Dromiciops bozinovici.* The function gene associated with the locus is in Table S7. The locus name corresponds to the ID locus in Table S7.

Figure S11. Major allele frequency for putative candidate loci identified by genome–environment association analyses using LFMM and RDA for *Dromiciops gliroides.* The function gene associated with the locus is in Table S8. The locus name corresponds to ID in locus Table S8.

Figure S12. Redundancy analysis (RDA) Redundancy analysis (RDA) using environment variables from WorldClim and elevation for **a)** *Dromiciops bozinovici* and **b)** *D. gliroides*. Only the first two axes are shown for each RDA. The direction of the main variation for each variables is indicated by a blue vector. Colored points represent localities, gray points are SNPs, and red points represent putative SNPs of selection most strongly correlated with variables in RDA.

Figure S13. Redundancy analysis (RDA) using environment variables from WorldClim and elevation for **a-b)** *D. gliroides without* clade DG-A *(Dromiciops mondaca).* Only the first two axes are shown for each RDA. Colored points represent localities, gray points are SNPs, and red points represent putative SNPs of selection most strongly correlated with variables in RDA. Venn diagram showing the number of loci identified as putatively under selection for *D. gliroides* without clade DG-A using two methods (LFFM and RDA).

Figure S14. Major allele frequency for putative candidate loci identified by genome–environment association analyses using LFMM and RDA for *Dromiciops gliroides.* without cluster DG-A. The function gene associated with the locus is in Table S9. The locus name corresponds to ID in locus Table S9.

Figure S15. Depiction of changes in minor allele frequencies of each putative candidate loci identified by genome–environment association analyses for *D. bozinovici*. Black circles reflect present frequencies, blue circles indicate future frequencies and horizontal lines indicate confidence. The locus name corresponds to the ID locus in Table S7. The name of the localities is on the left side, based on Table 1.

Figure S16. Depiction of changes in allele frequencies in geographical space via AlleleShift candidate loci identified by genome–environment association for *D. bozinovici* in the future. Triangle colors for the future frequencies indicate trends (red to decreasing, green to increasing). The locus name corresponds to the ID locus in Table S7.

Figure S17. Depiction of changes in minor allele frequencies of each putative candidate loci identified by genome–environment association analyses for *D. gliroides.* Black circles reflect present frequencies, blue circles indicate future frequencies and horizontal lines indicate confidence. The locus name corresponds to the ID locus in Table S8. The name of the localities is on the left side, based on Table 1.

Figure S18. Depiction of changes in allele frequencies in geographical space via AlleleShift candidate loci identified by genome–environment association analyses for *D. gliroides* in the future. Triangle colors for the future frequencies indicate trends (red to decreasing, green to increasing).The locus name corresponds to the ID locus in Table S8.

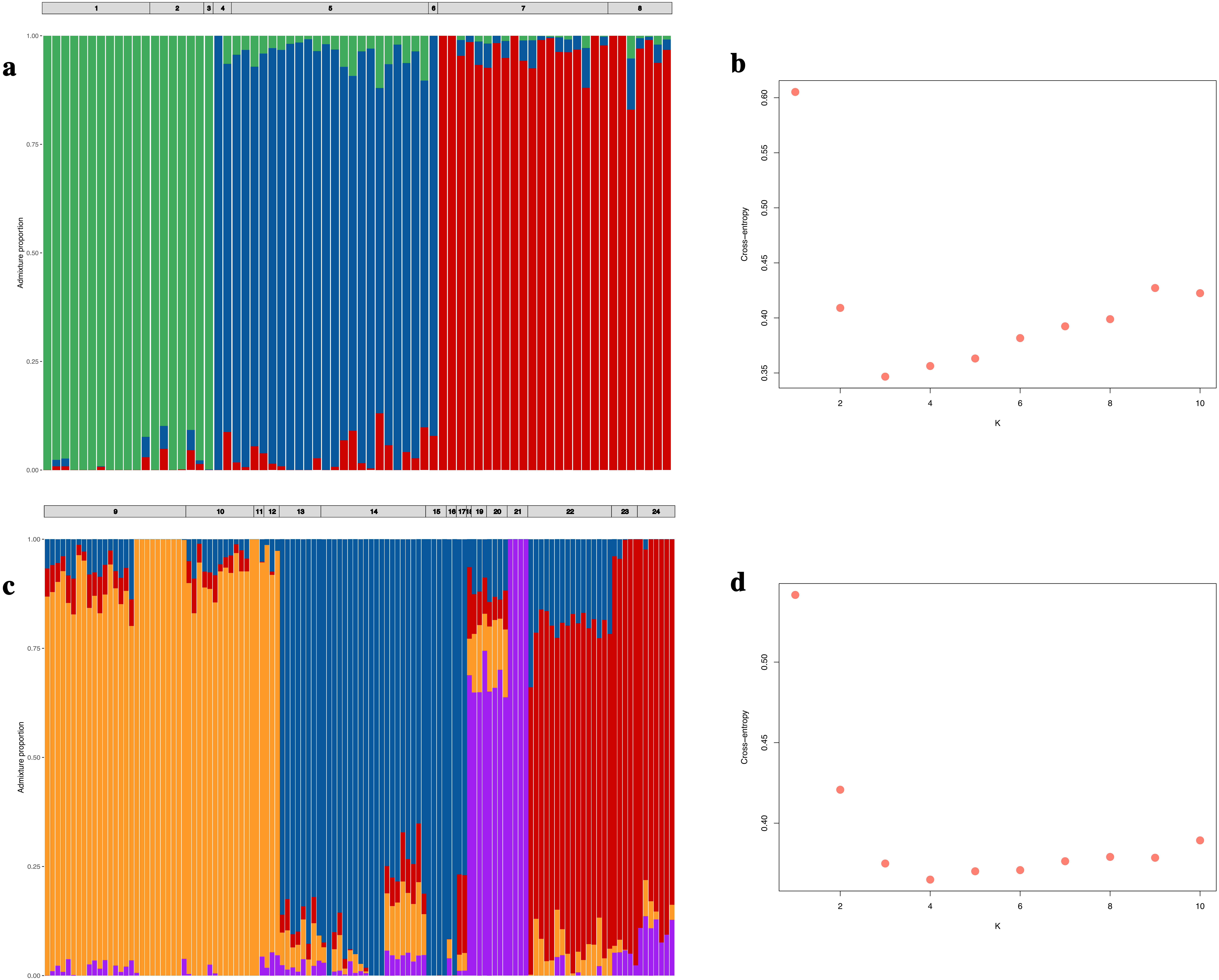
a
b
c
d
1 2 3 4 5 6 7 8
9 10 11 12 13 14 15 16 17 19 20 21 22 23 24
Admixture proportion
0.00
0.25
0.50
0.75
1.00
Cross−entropy
0.35
0.40
0.45
0.50
0.55
0.60
K
2
4
6
8
10
Fig S1

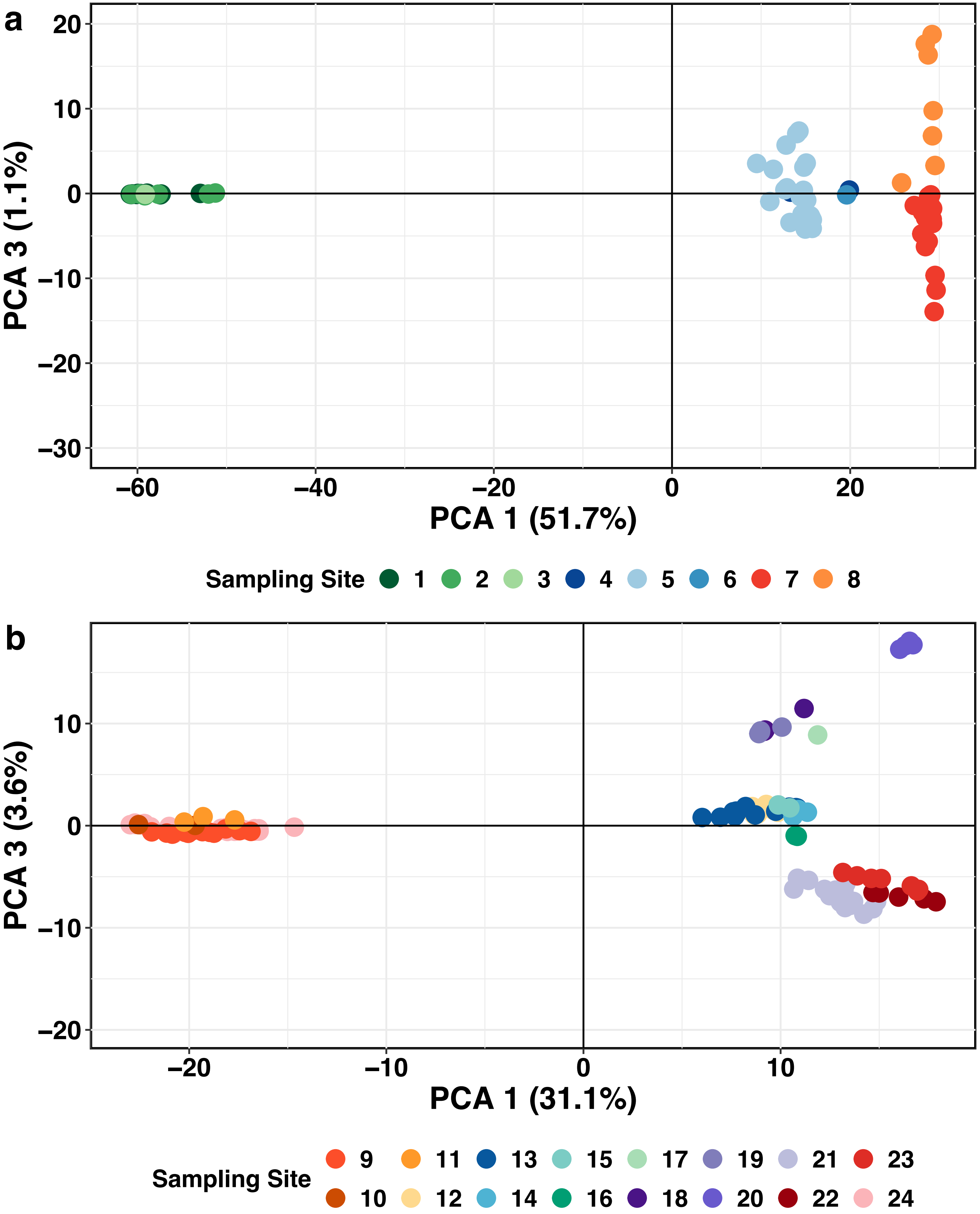
a
PCA 3 (1.1%)
PCA 1 (51.7%)
Sampling Site 1 2 3 4 5 6 7 8
b
PCA 3 (3.6%)
PCA 1 (31.1%)
Sampling Site 9 10 11 12 13 14 15 16 17 18 19 20 21 22 23 24

Fig S2

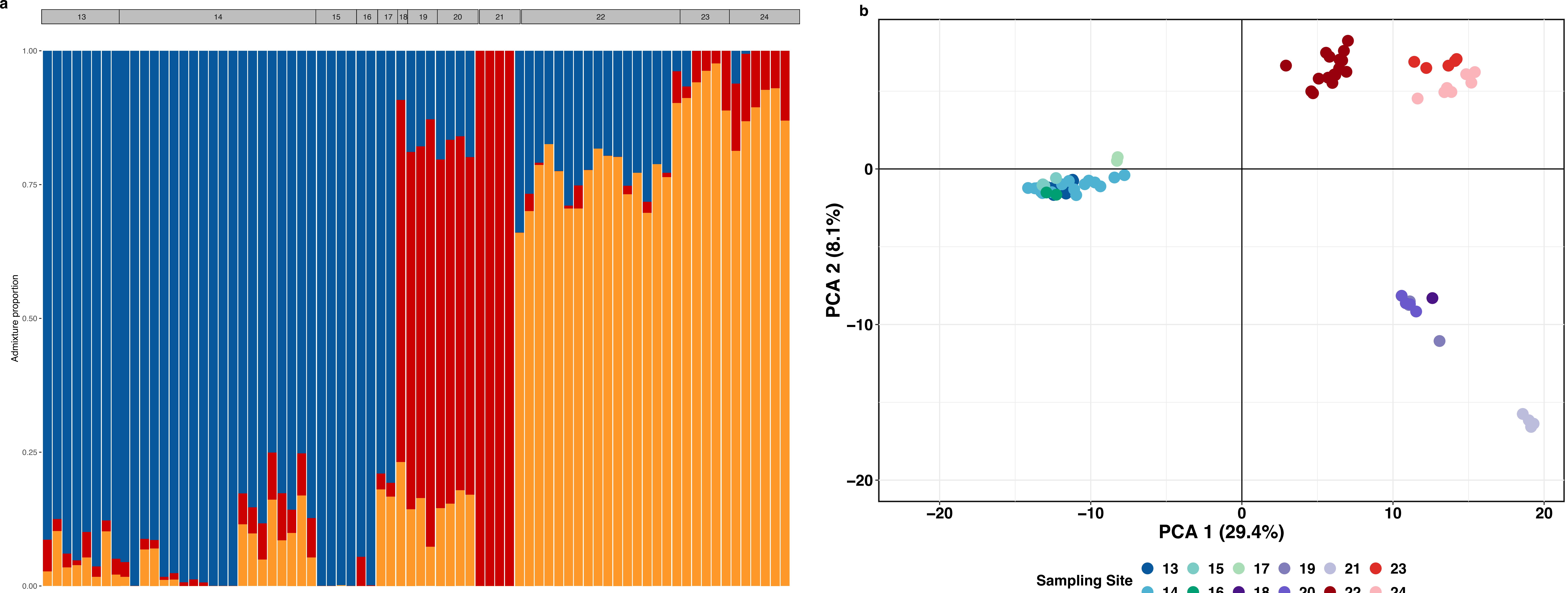
a
13
14
15
16
17
18
19
20
21
22
23
24
Admixture proportion
1.00
0.75
0.50
0.25
0.00
b
PCA 2 (8.1%)
PCA 1 (29.4%)
0
−10
−20
10
20
Sampling Site
13
14
15
16
17
18
19
20
21
22
23
24

Fig S3

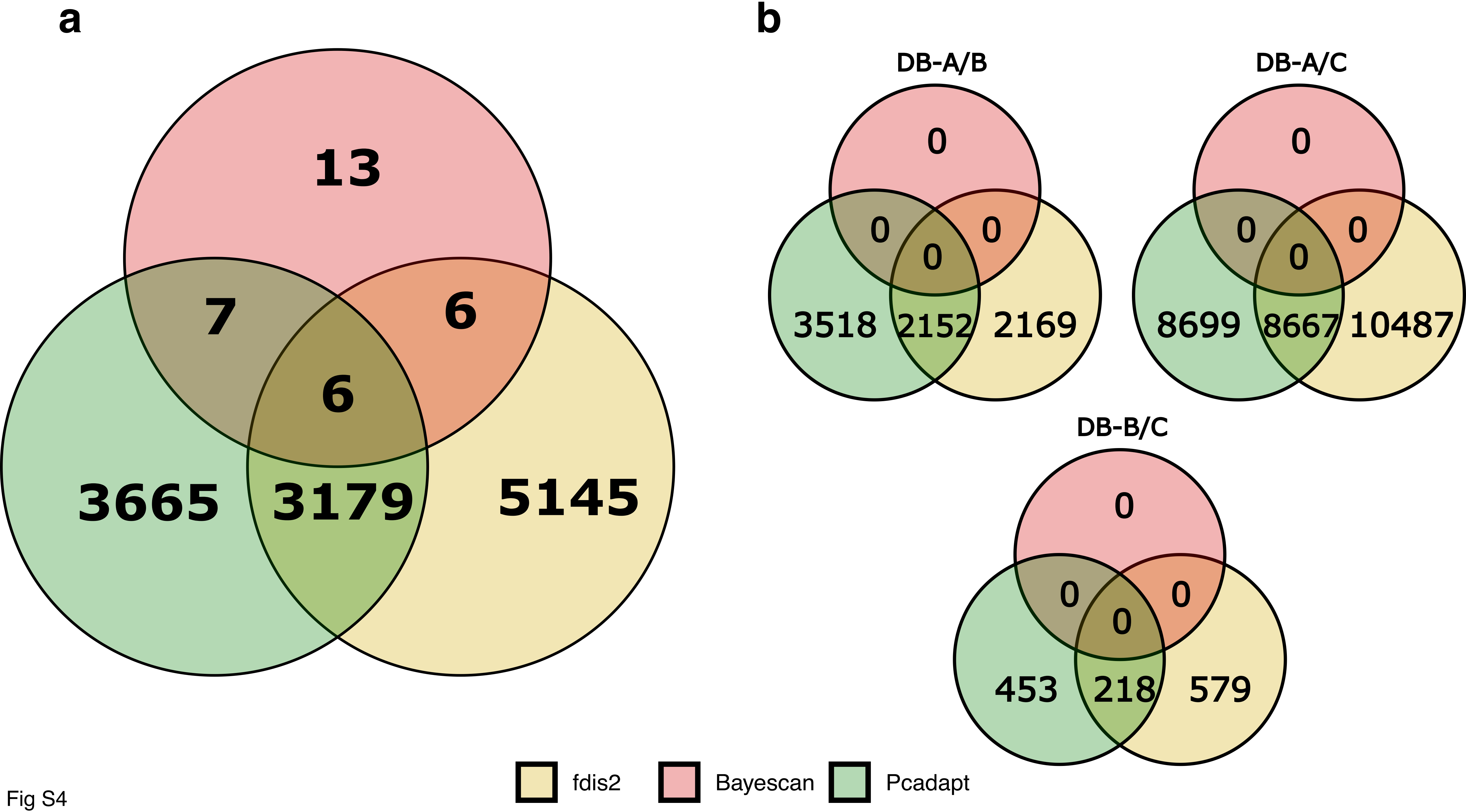
a
13
7
6
6
3665
3179
5145
b
DB-A/B
0
0
0
0
3518
2152
2169
DB-A/C
0
0
0
0
8699
8667
10487
DB-B/C
0
0
0
0
453
218
579
fdis2
Bayescan
Pcadapt
Fig S4

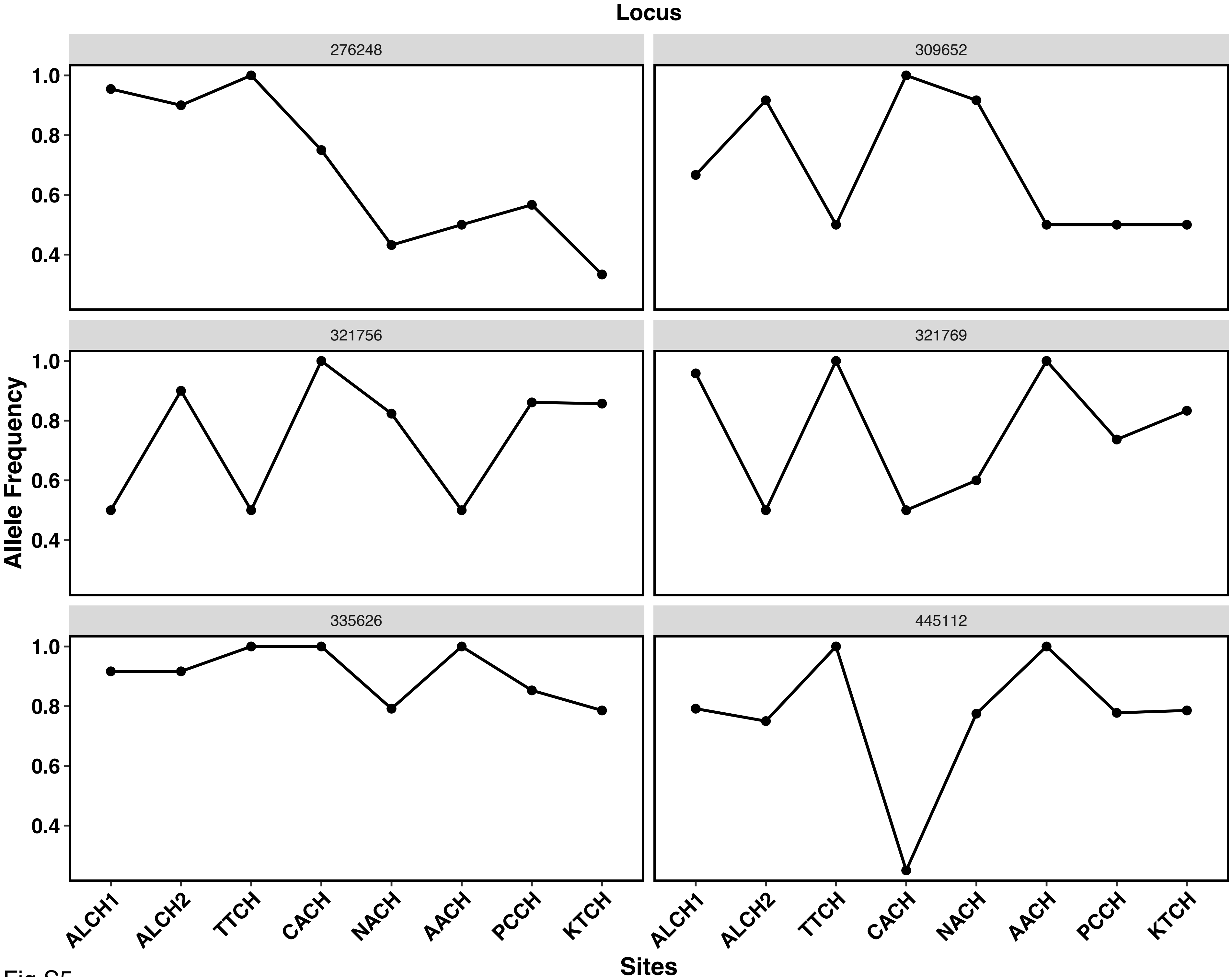
Locus
276248
309652
321756
321769
335626
445112
Allele Frequency
1.0
0.8
0.6
0.4
ALCH1
ALCH2
TTCH
CACH
NACH
AACH
PCCH
KTCH
Sites
Fig S5

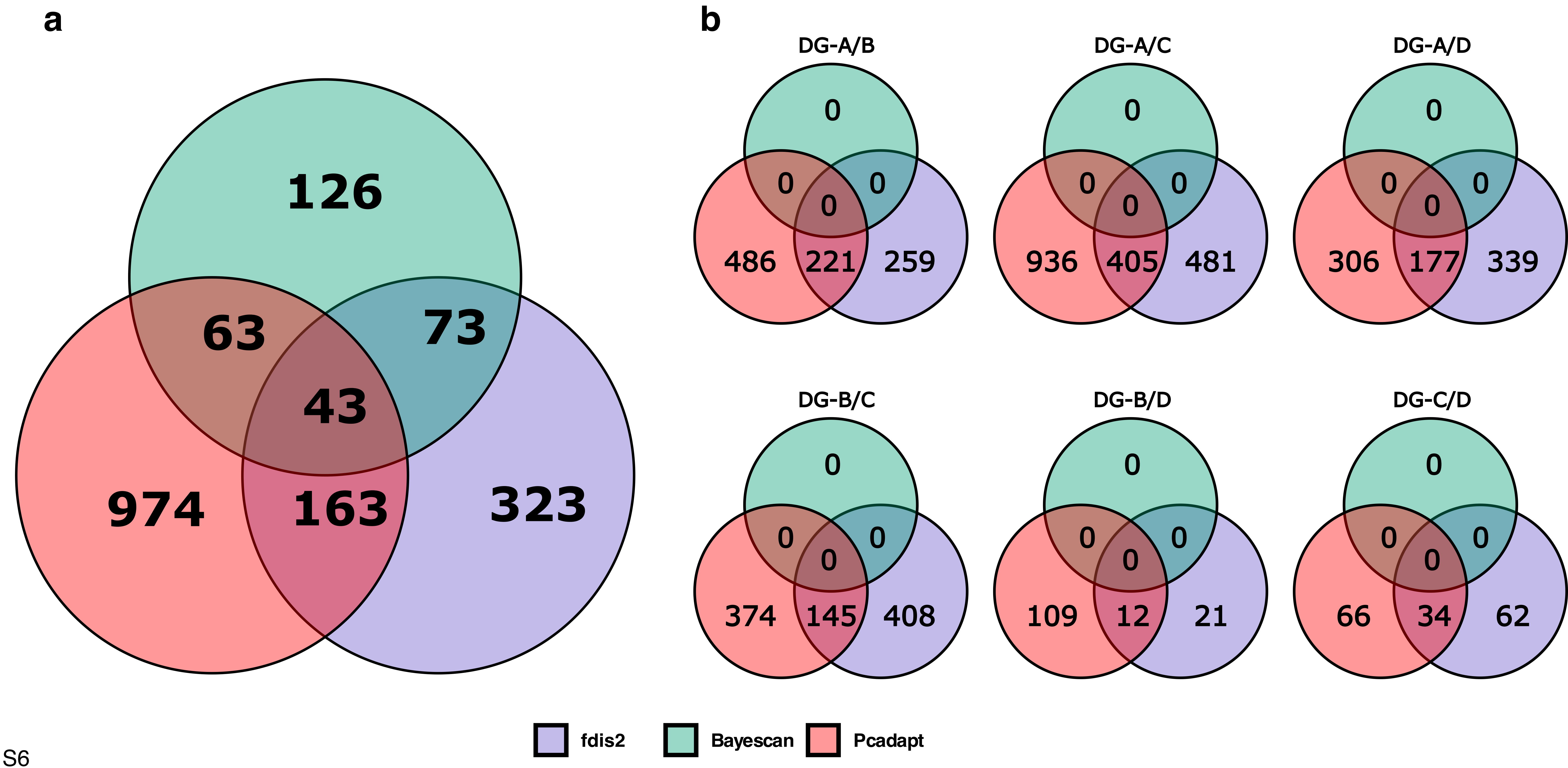
a
126
63
73
43
974
163
323
b
DG-A/B
0
0
0
0
486
221
259
DG-A/C
0
0
0
0
936
405
481
DG-A/D
0
0
0
0
306
177
339
DG-B/C
0
0
0
0
374
145
408
DG-B/D
0
0
0
0
109
12
21
DG-C/D
0
0
0
0
66
34
62
fdis2
Bayescan
Pcadapt

Fig S6

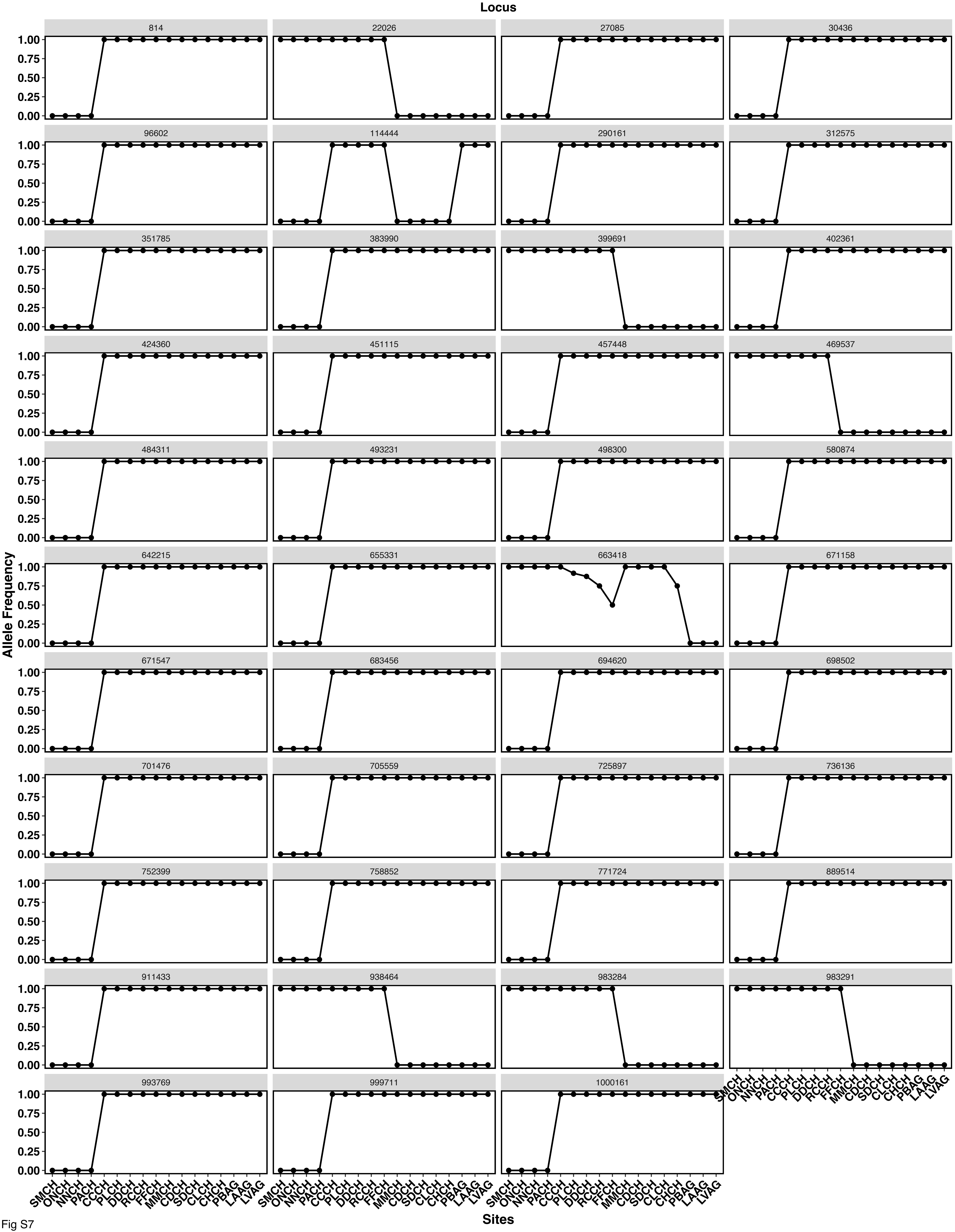
Locus
814
22026
27085
30436
96602
114444
290161
312575
351785
383990
399691
402361
424360
451115
457448
469537
484311
493231
498300
580874
642215
655331
663418
671158
671547
683456
694620
698502
701476
705559
725897
736136
752399
758852
771724
889514
911433
938464
983284
983291
993769
999711
1000161
Allele Frequency
1.00
0.75
0.50
0.25
0.00
SMCH ONCH NNCH PACH CCCH PLCH DDCH RCCH FFCH MMCH CDCH SDCH CLCH CHCH PBAG LAAG LVAG
Sites

Fig S7

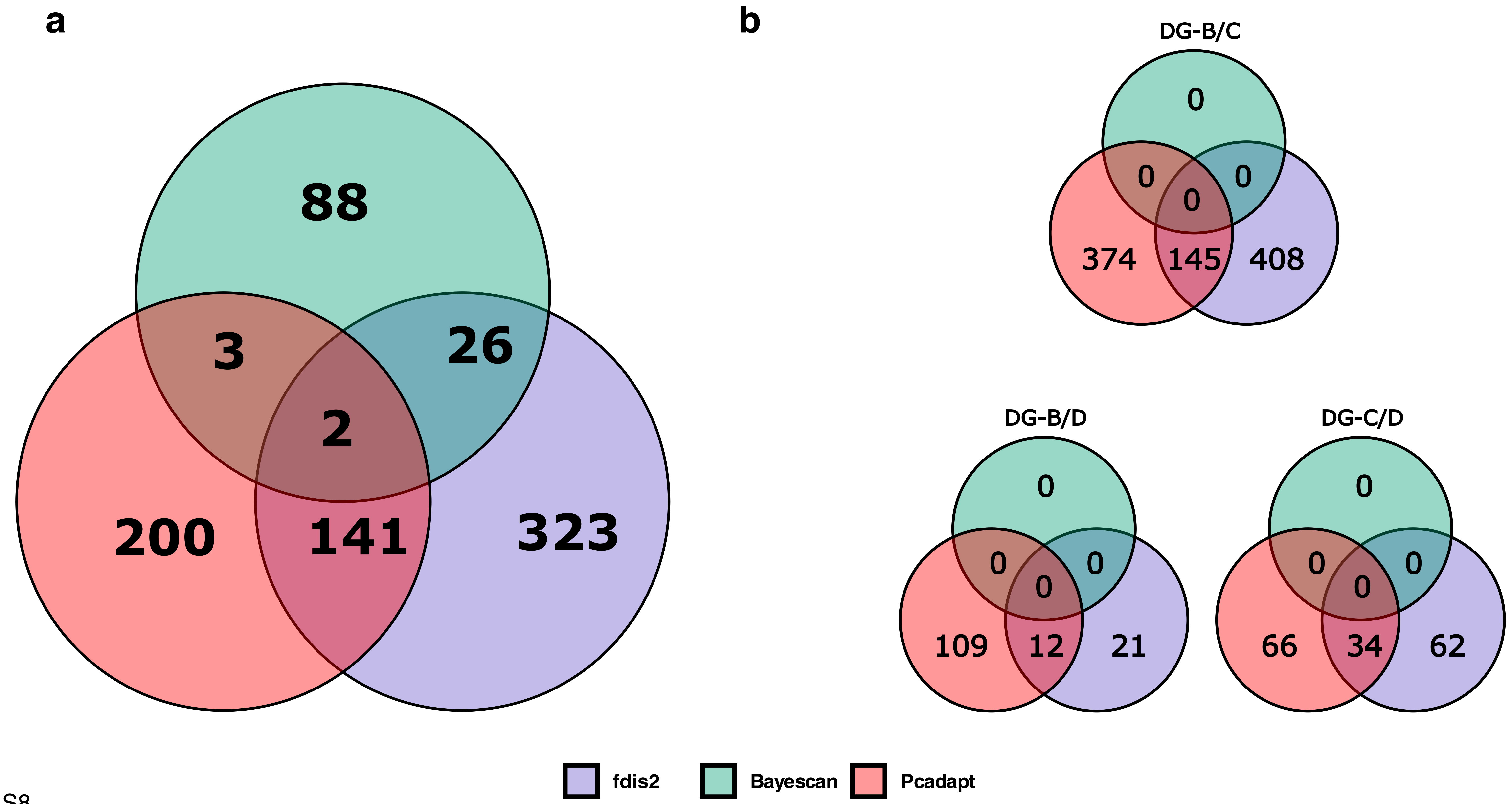
a
88
3
26
2
200
141
323
b
DG-B/C
0
0
0
0
374
145
408
DG-B/D
0
0
0
0
109
12
21
DG-C/D
0
0
0
0
66
34
62
fdis2
Bayescan
Pcadapt

Fig S8

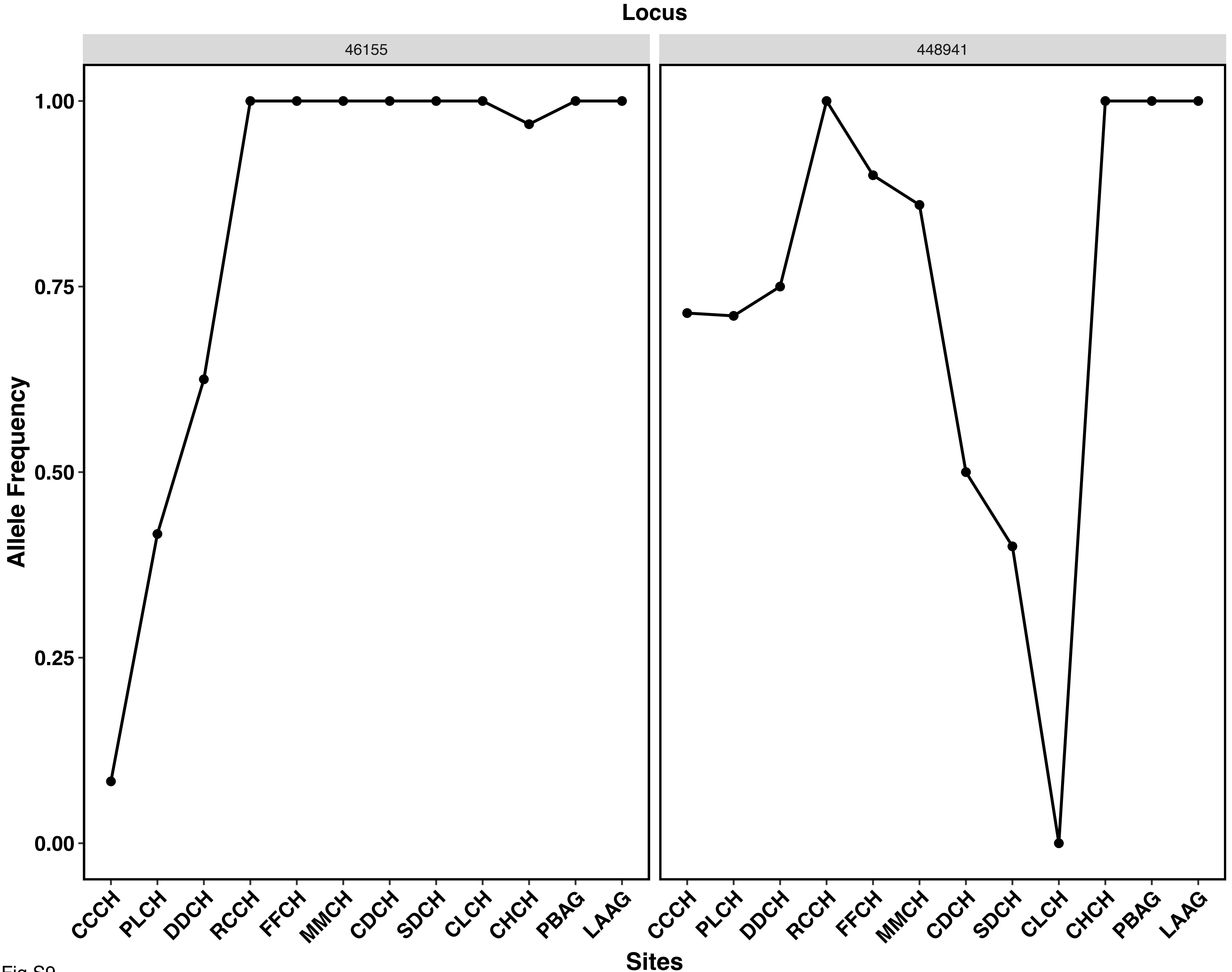

Locus
46155
448941
Allele Frequency
1.00
0.75
0.50
0.25
0.00
CCCH
PLCH
DDCH
RCCH
FFCH
MMCH
CDCH
SDCH
CLCH
CHCH
PBAG
LAAG
Sites

Fig S9

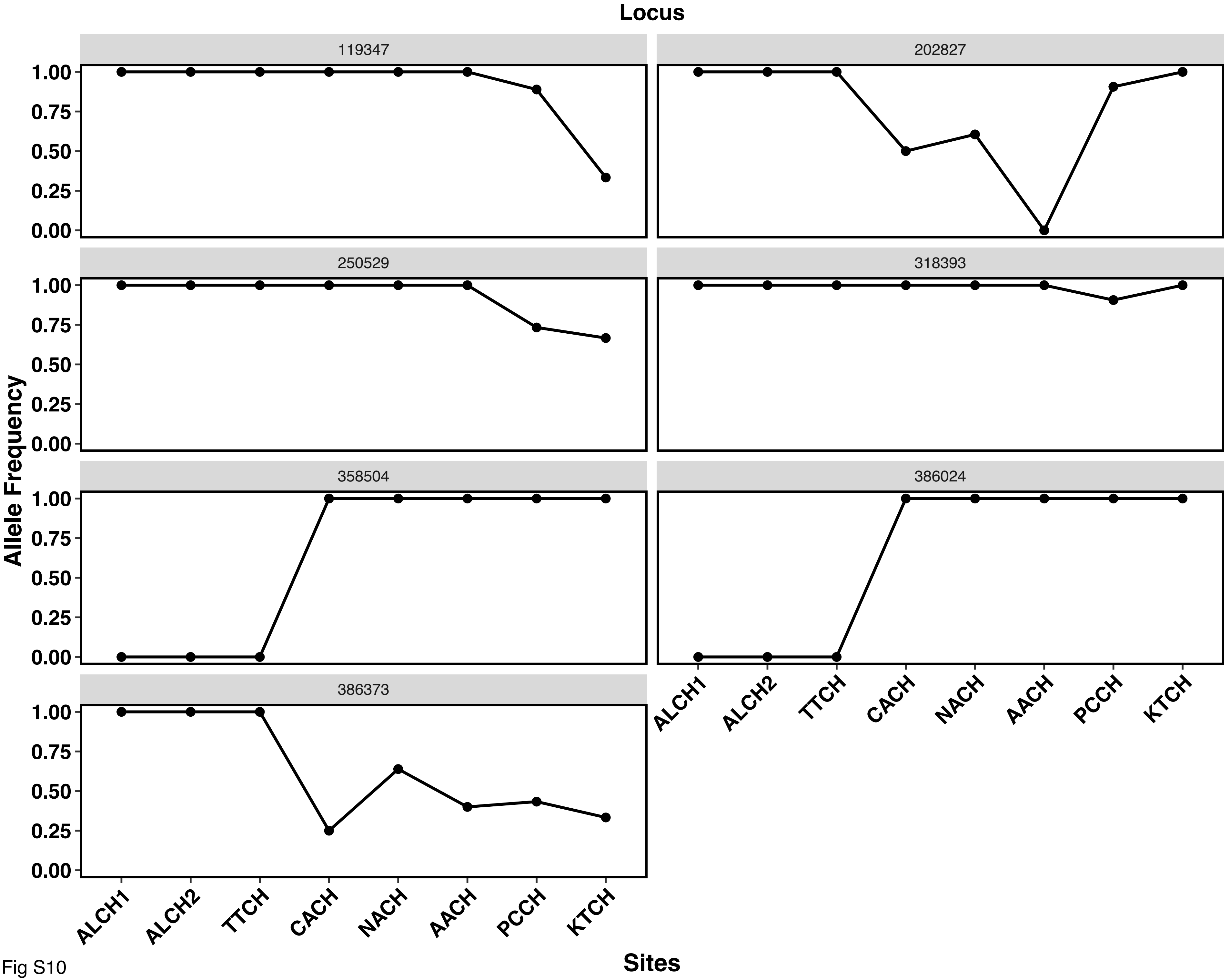
Locus
119347
202827
250529
318393
358504
386024
386373
1.00
0.75
0.50
0.25
0.00
Allele Frequency
ALCH1
ALCH2
TTCH
CACH
NACH
AACH
PCCH
KTCH
Sites

Fig S10

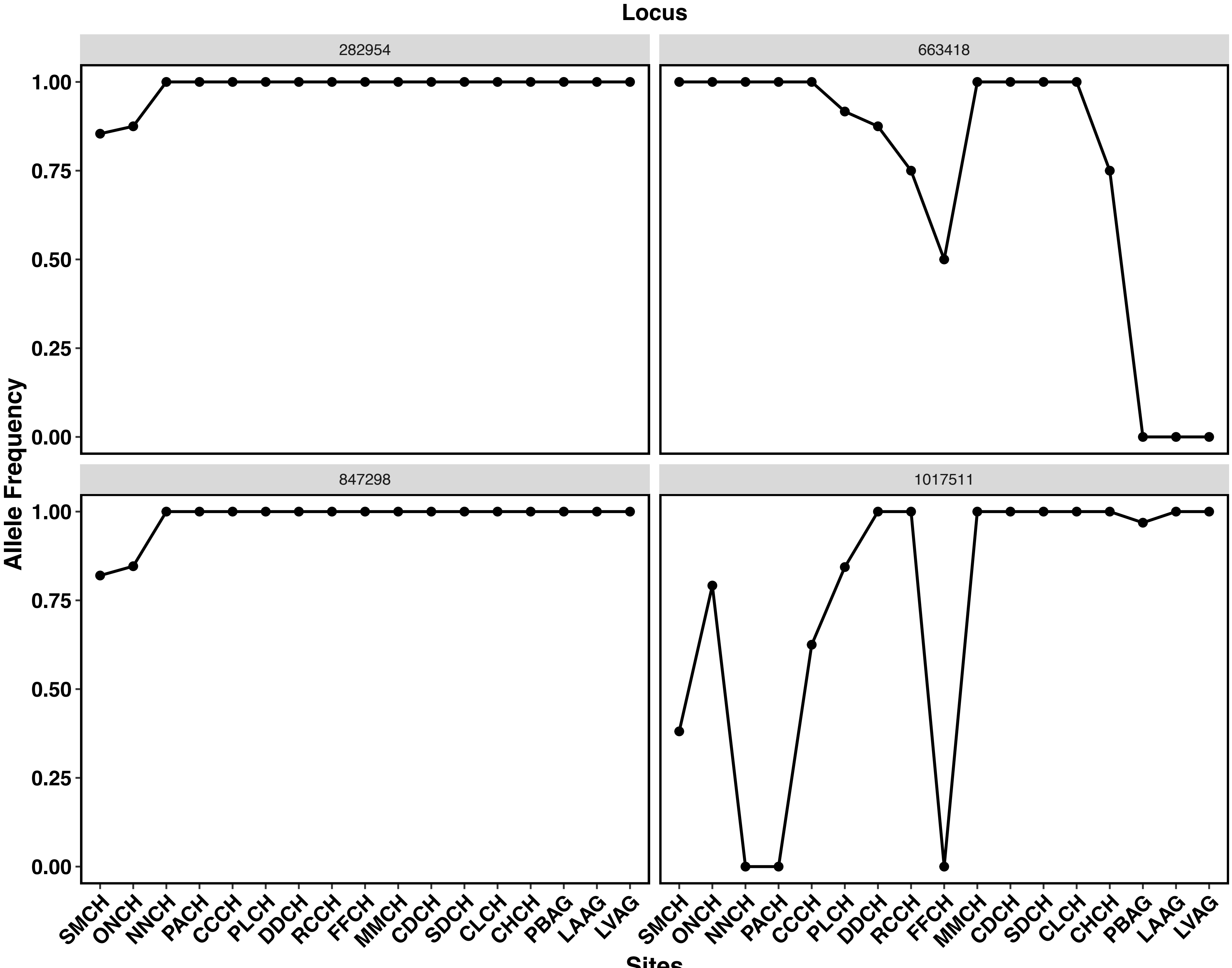

Locus
282954
663418
847298
1017511
Allele Frequency
0.00
0.25
0.50
0.75
1.00
SMCH
ONCH
NNCH
PACH
CCCH
PLCH
DDCH
RCCH
FFCH
MMCH
CDCH
SDCH
CLCH
CHCH
PBAG
LAAG
LVAG
Sites

Fig S11

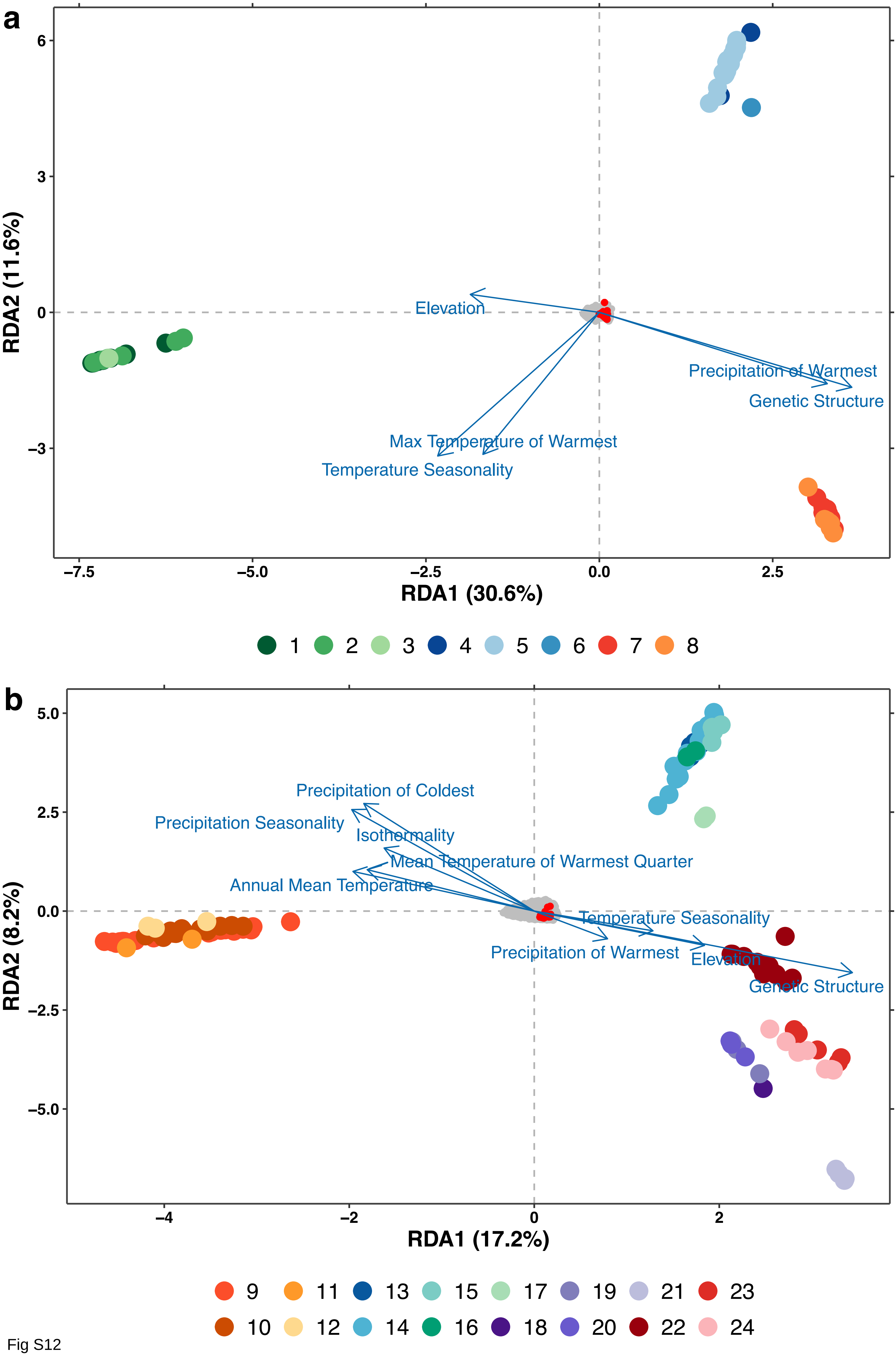

a
RDA2 (11.6%)
RDA1 (30.6%)
6
3
0
−3
−7.5
−5.0
−2.5
0.0
2.5
Elevation
Precipitation of Warmest
Genetic Structure
Max Temperature of Warmest
Temperature Seasonality
1
2
3
4
5
6
7
8
b
RDA2 (8.2%)
RDA1 (17.2%)
5.0
2.5
0.0
−2.5
−5.0
−4
−2
0
2
Precipitation of Coldest
Precipitation Seasonality
Isothermality
Mean Temperature of Warmest Quarter
Annual Mean Temperature
Temperature Seasonality
Precipitation of Warmest
Elevation
Genetic Structure
9
10
11
12
13
14
15
16
17
18
19
20
21
22
23
24

Fig S12

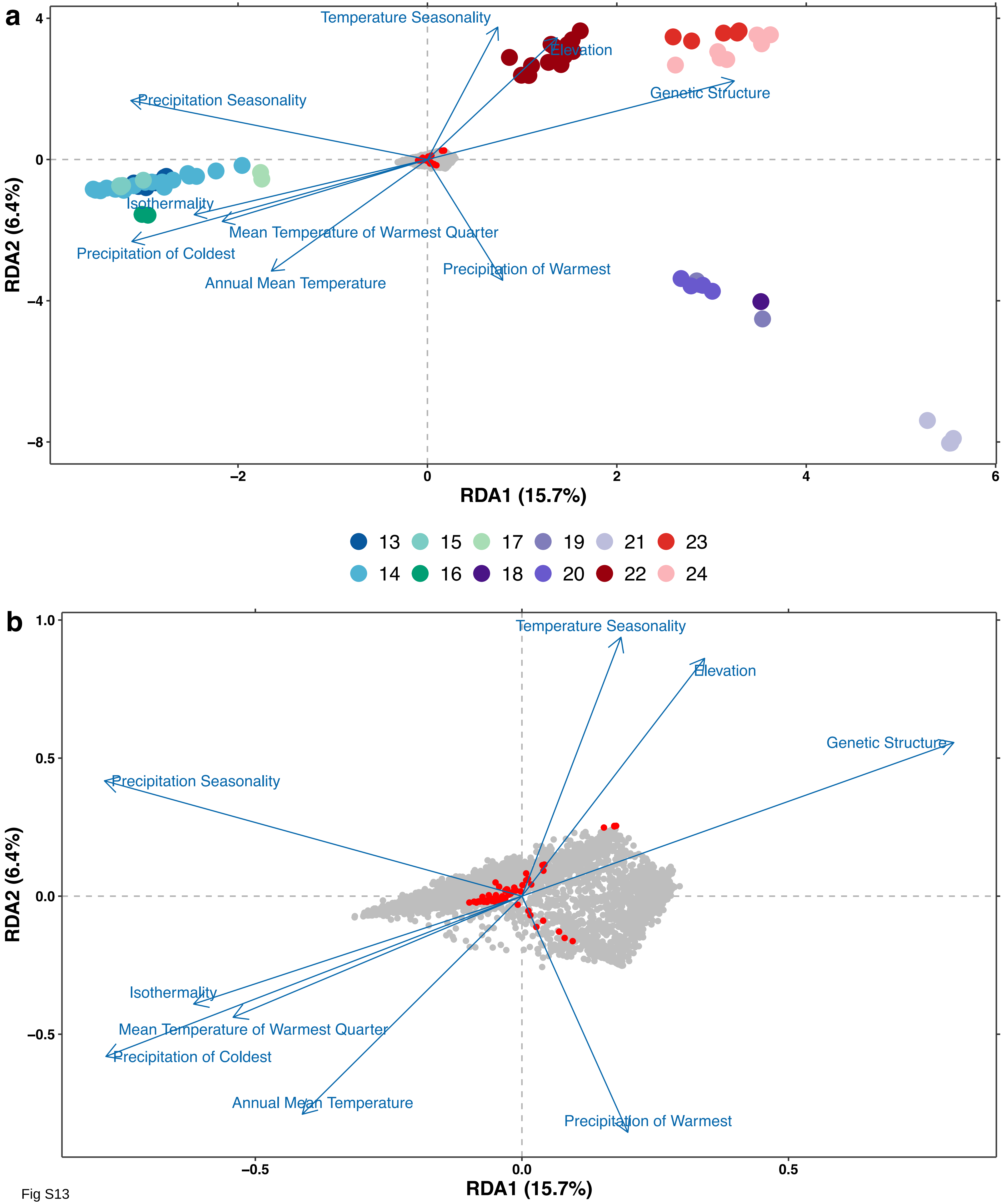
a
Temperature Seasonality
Elevation
Genetic Structure
Precipitation Seasonality
Isothermality
Mean Temperature of Warmest Quarter
Precipitation of Coldest
Annual Mean Temperature
Precipitation of Warmest
RDA1 (15.7%)
RDA2 (6.4%)
13 15 17 19 21 23
14 16 18 20 22 24
b
Temperature Seasonality
Elevation
Genetic Structure
Precipitation Seasonality
Isothermality
Mean Temperature of Warmest Quarter
Precipitation of Coldest
Annual Mean Temperature
Precipitation of Warmest
RDA1 (15.7%)
RDA2 (6.4%)

Fig S13

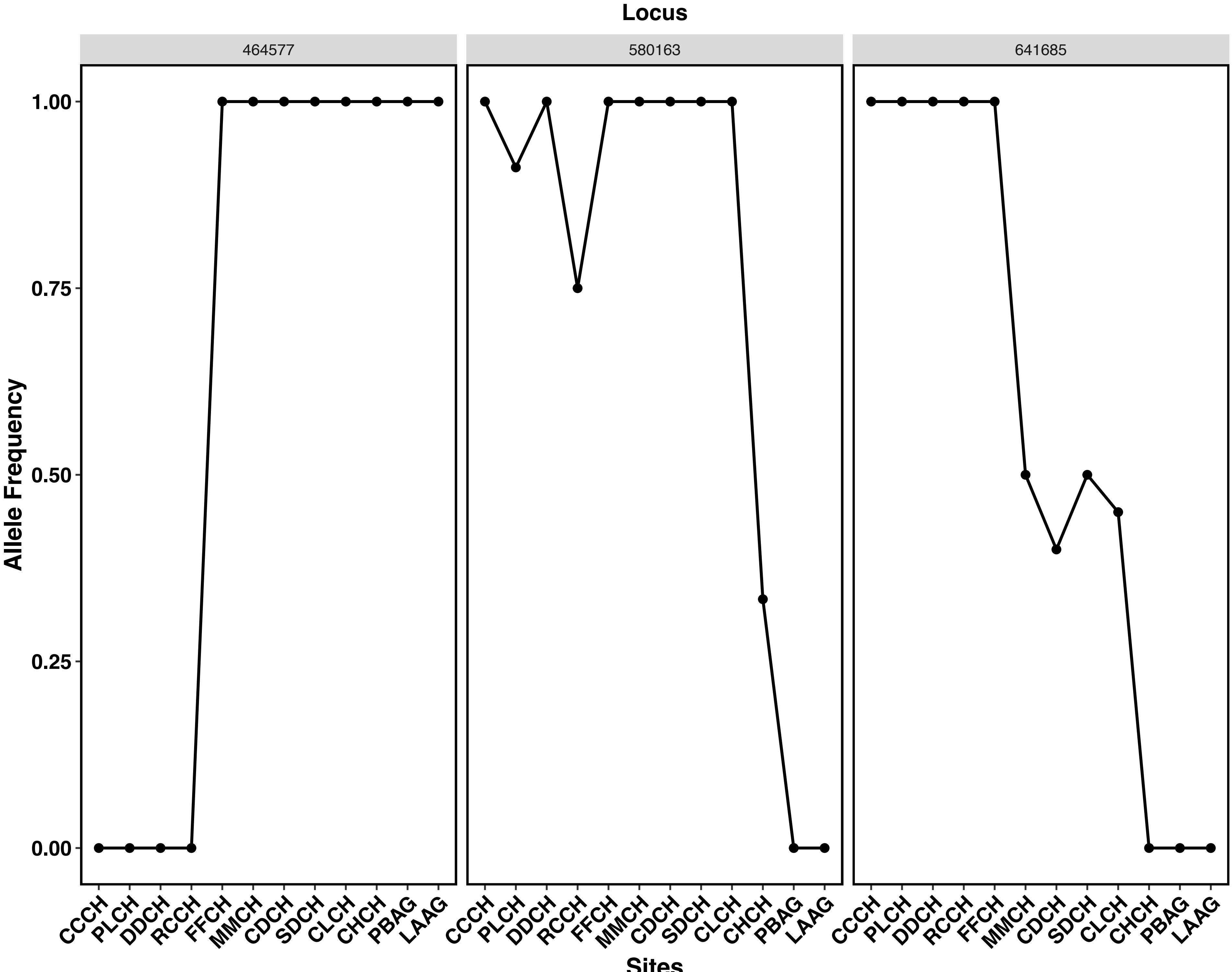

Locus
464577
580163
641685
1.00
0.75
0.50
0.25
0.00
Allele Frequency
CCCH
PLCH
DDCH
RCCH
FFCH
MMCH
CDCH
SDCH
CLCH
CHCH
PBAG
LAAG
Sites

Fig S14

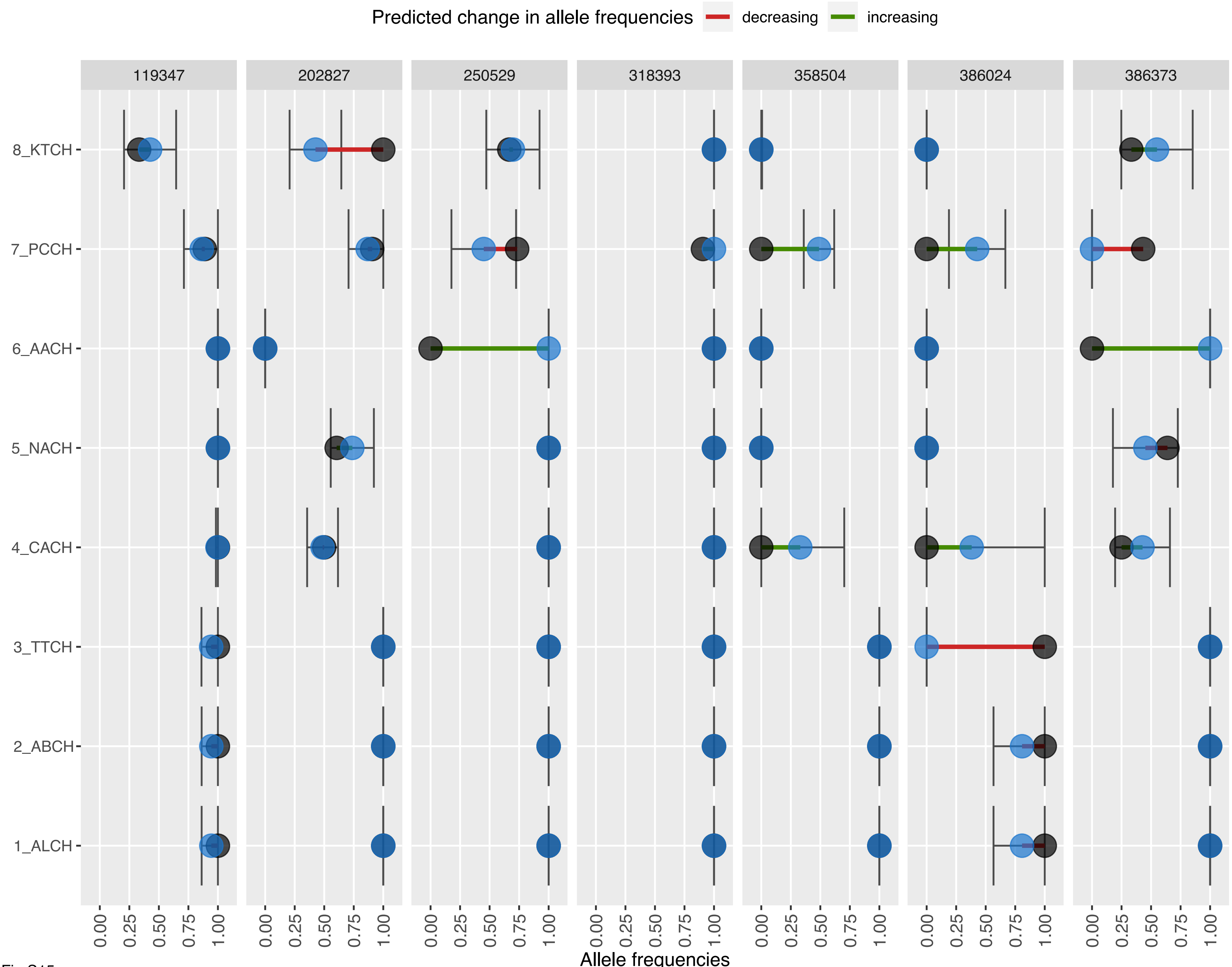
Predicted change in allele frequencies
decreasing
increasing
119347
202827
250529
318393
358504
386024
386373
8_KTCH
7_PCCH
6_AACH
5_NACH
4_CACH
3_TTCH
2_ABCH
1_ALCH
0.00
0.25
0.50
0.75
1.00
Allele frequencies

Fig S15

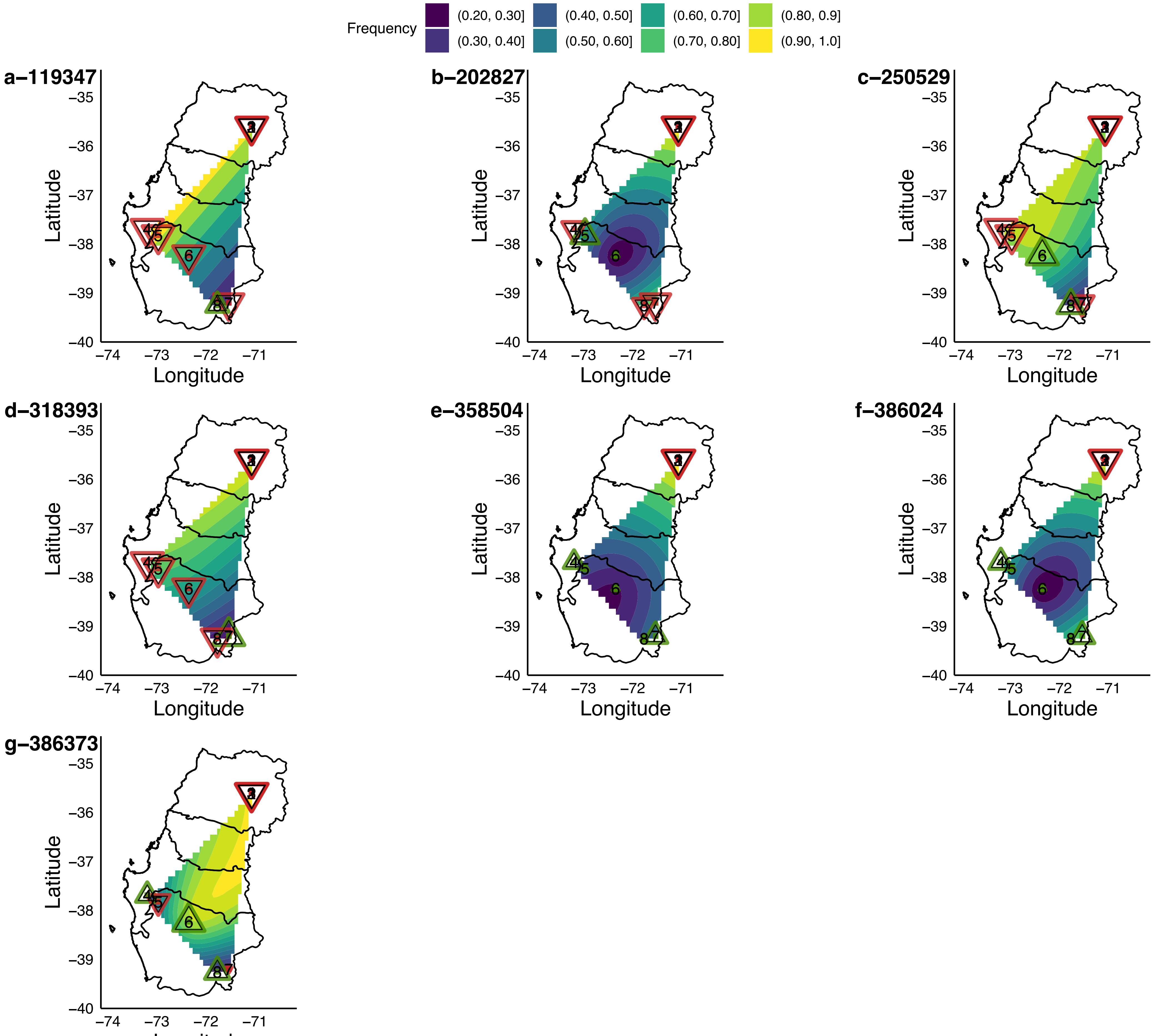
Frequency
(0.20, 0.30]
(0.30, 0.40]
(0.40, 0.50]
(0.50, 0.60]
(0.60, 0.70]
(0.70, 0.80]
(0.80, 0.9]
(0.90, 1.0]
a–119347
b–202827
c–250529
d–318393
e–358504
f–386024
g–386373
Latitude
Longitude

Fig S16

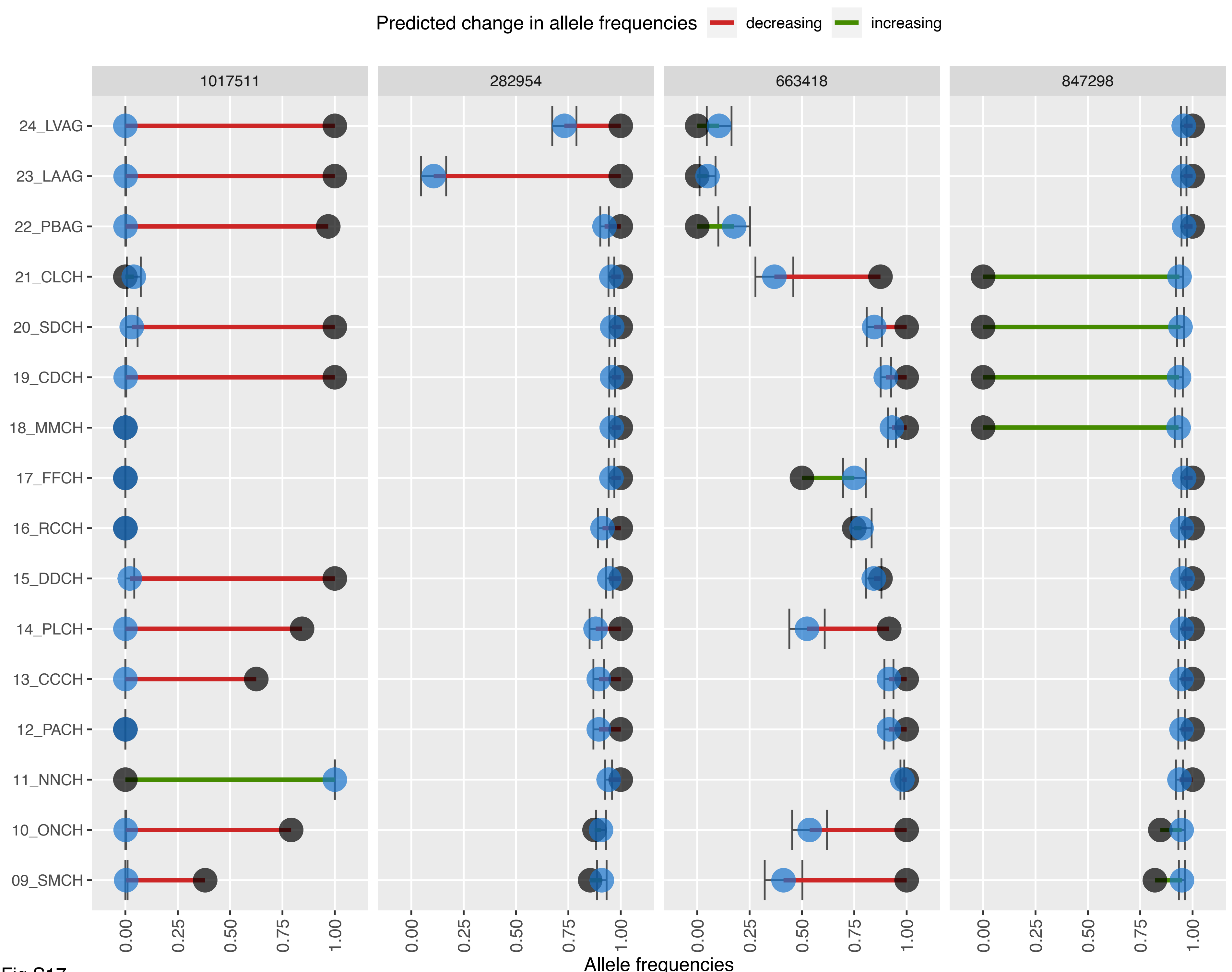

Predicted change in allele frequencies
decreasing
increasing
1017511
282954
663418
847298
24_LVAG
23_LAAG
22_PBAG
21_CLCH
20_SDCH
19_CDCH
18_MMCH
17_FFCH
16_RCCH
15_DDCH
14_PLCH
13_CCCH
12_PACH
11_NNCH
10_ONCH
09_SMCH
0.00
0.25
0.50
0.75
1.00
Allele frequencies

Fig S17

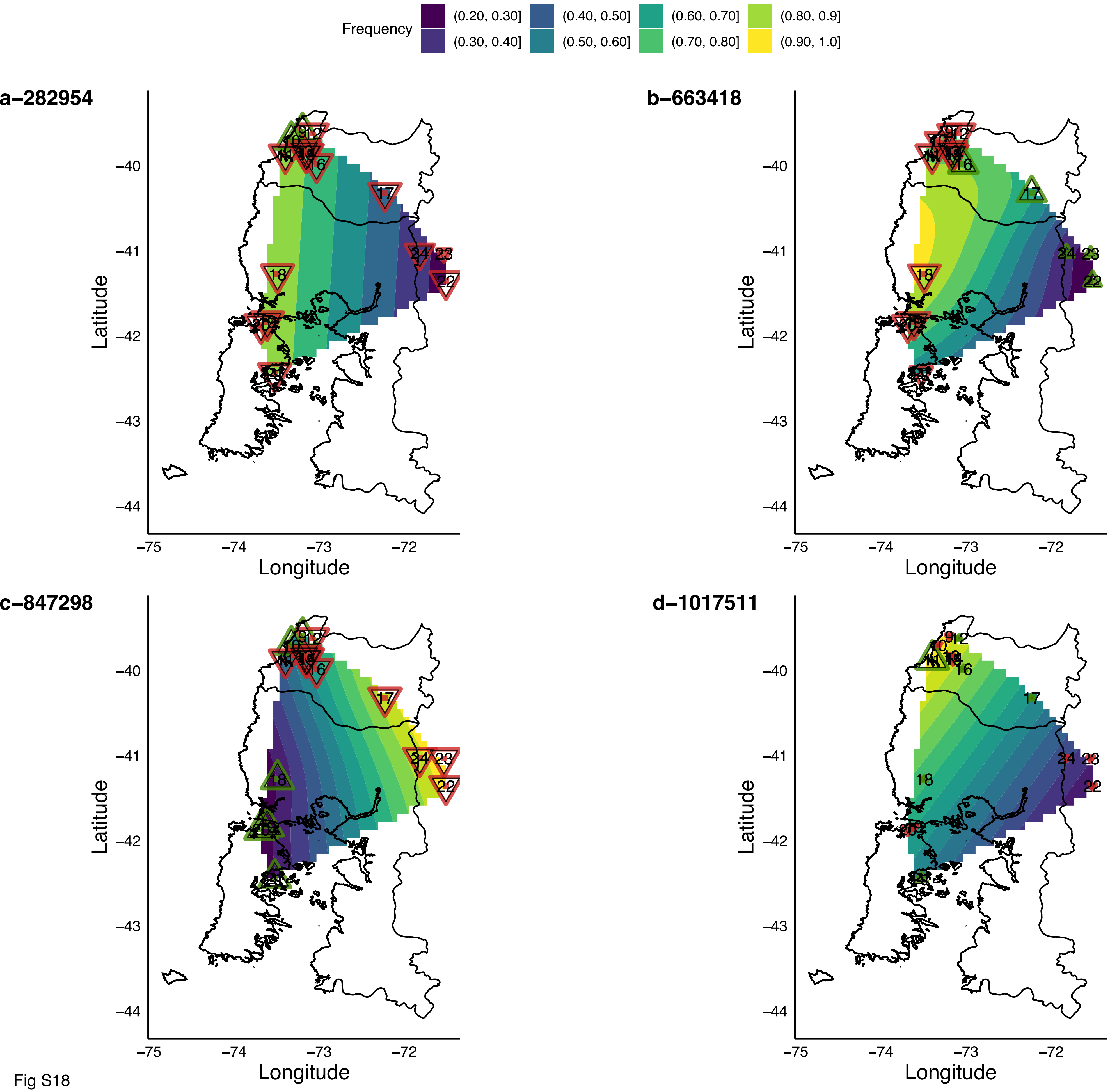
Frequency
(0.20, 0.30]
(0.30, 0.40]
(0.40, 0.50]
(0.50, 0.60]
(0.60, 0.70]
(0.70, 0.80]
(0.80, 0.9]
(0.90, 1.0]
a−282954
b−663418
c−847298
d−1017511
Latitude
Longitude
−40
−41
−42
−43
−44
−75
−74
−73
−72

Fig S18
